# DNA‐Binding Properties of Iridium(III) Complexes Encompassing Design, Function and Biophysical Assessments

**DOI:** 10.1002/cphc.70426

**Published:** 2026-06-05

**Authors:** Ibrahim S. Alkhaibari, Niklaas J. Buurma, Simon J. A. Pope

**Affiliations:** ^1^ School of Chemistry Cardiff University Cardiff UK; ^2^ Qassim University Buraydah Saudi Arabia

**Keywords:** biophysical chemistry, cellular imaging, chemistry, DNA binding agent, iridium, ligand, luminescence, molecular probe

## Abstract

The increasingly diverse biological applications of iridium(III) complexes, from luminescent cellular imaging agents to therapeutic and prospective theranostics, has been enabled by a combination of imaginative ligand design and tunable (photo)physical properties. Within this biological context, it is crucial to understand how molecular probes interact with biological substrates and macromolecules. This review focuses upon the structural diversity of DNA‐binding metal complexes based upon iridium(III), the general workflow used to interrogate these interactions with DNA, and critically analyses the relationship between complex structure and functionality.

## Graphical abstract

This review describes the biophysical techniques used to investigate DNA‐binding phenomena and the progress made so far in the application of iridium(III) complexes for this purpose. Ir(III) complexes offer myriad opportunities in the design and study of DNA‐binding agents and discussed examples are contextualised with complementary studies in bioimaging and cytotoxicity.

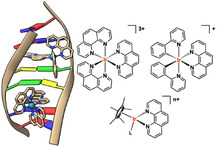

## 1 Introduction

The nature of the interactions between deoxyribonucleic acid (DNA) and metal‐ligand coordination complexes has been investigated for many decades. From fundamental studies on the precise description of these molecular interactions, through to the application areas where such interactions can be controlled and even exploited, metal complexes continue to provide unique opportunities for researchers across chemistry and biology research disciplines. Many reviews of the literature have appeared over the last 20 years that describe various aspects of metal complex DNA interactions [1, 2, 3, 4, 5, 6, 7, 8]. Metal complexes are particularly attractive because of the control and structural variety offered by different metal ion and ligand combinations. Positive charge, steric and geometric control, the use of planar hydrophobic ligand regions, and labile ligand sites can all be designed to promote different types of DNA interactions. Tunable redox character and optical and luminescent properties are also desirable providing a wide array of approaches to study and characterise the nature of DNA interactions.

Two categories of binding mode can occur between metal complexes and DNA: reversible or irreversible [2]. The latter typically involves covalent bond formation, and more specifically a coordinate bond, between the DNA and a metal ion and usually implies ligand substitution at the metal centre (e.g. cisplatin where hydrolysis of the chloride ligands facilitates coordination of a purine base within DNA). Reversible DNA binding is typically non‐covalent and encompasses electrostatic, intercalation and groove (major or minor) binding modes. Metal complexes are especially attractive at addressing the different design criteria associated with these varied binding types. Given the comprehensive nature of previous reviews across this topic [3, 4, 5, 6, 7, 8], the focus of this current article is two‐fold: i) a contextual overview of accessible state‐of‐the‐art techniques used to interrogate molecular interactions with DNA; ii) the utility of iridium(III) based coordination complexes, in their various guises, as DNA binders.

## 2 Biological Applicability of Iridium(III) Complexes

**FIGURE 1:**
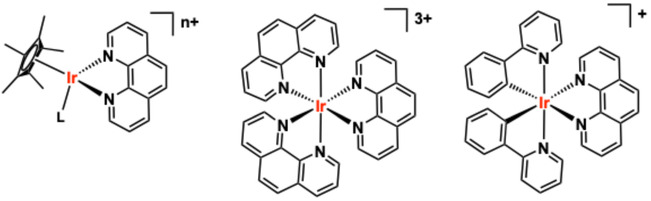
The basic structures of the three classes of Ir(III) complex (each using 1,10‐phenantholine as a (co‐)ligand) discussed within the review. Left‐to‐right: half‐sandwich (piano‐stool) metallocene; octahedral polypyridine; octahedral cyclometalated.

Metal‐based compounds have long contributed to medical advances, either as therapeutics (e.g. cisplatin in DNA‐targeted chemotherapy) [9], diagnostics in magnetic resonance imaging and radioimaging, or as cellular imaging agent for fluorescence microscopy, or ultimately, as smart theranostic agents [10, 11]. In this context, Ir(III) complexes (particularly, and most commonly, organometallic variants) have attracted significant attention [12]. A variety of Ir(III) complexes have emerged as promising candidates due to their ease of structural tunability (via ligands), ability to engage DNA in selective and controllable ways [13, 14, 15], and their photoluminescent properties [16]. There are three predominant classes of Ir(III) complex that have been studied in this context and are thus considered herein: i) half‐sandwich, “piano stool” Ir(III) complexes; ii) octahedral polypyridine complexes; and iii) octahedral cyclometalated Ir(III) species (Figure 1). The contrasting properties and reactivities of these different classes of molecule have dictated their types of biological application. Many “piano stool” Ir(III) complexes have been studied in terms of cytotoxicity and anticancer activity [17] but generally lack useful photophysical attributes for non‐invasive optical imaging experiments [18]. In contrast, octahedral cyclometalated Ir(III) species that are prevalent in optical imaging studies because of their favourable luminescent properties and excellent photostability [19] are now being intensely studied regarding targeted modes [20, 21, 22, 23] of biological action through cytotoxic [24] and phototoxic (with opportunities in photodynamic therapy [25]) properties [26].

The advantages offered by Ir(III) complexes [27, 28, 29, 30, 31, 32, 33, 34] include the ease of tuning or adapting the ligands, which can be added in a stepwise manner, to control important physical properties such as solubility and charge, as well as the inherent photophysical attributes [35, 36] that can allow luminescence wavelengths into the deep red region [37, 38, 39, 40, 41, 42, 43, 44, 45]. These amenable design features, married with kinetic inertness and high photostability, have (re)positioned functional organometallic Ir(III) complexes as viable options within the life sciences [46, 47, 48, 49, 50, 51, 52]. Over the last 10–15 years, numerous studies have highlighted the attractive features of octahedral cyclometalated Ir(III) species that can be adapted for biological studies via fluorescence microscopy (and its time‐resolved, lifetime mapping variants, FLIM/PLIM) [53, 54, 55].

These attributes are particularly useful for studying the fundamental way in which Ir(III) complexes interact with common biomolecules, such as DNA [56]. This becomes especially relevant when the intracellular targets are the nucleus or mitochondria [57], both of which contain DNA, and when one considers the phototoxicity of Ir(III) species [58, 59]. Given the well‐established precedent for applying these complexes to tackle biological problems, including at a cellular level, it follows that it is undoubtedly important to fully understand how such species interact with endogenous (macro)molecules [60, 61]. It is important to note that even in the absence of chiral ligands, the octahedral Ir(III) complexes (Figure 1) can be optical isomers (Δ and Λ)—the different enantiomers can have different interactions with DNA.

Understanding how these complexes bind to DNA is crucial for designing next‐generation therapeutics and diagnostics [62, 63]. This review focuses on the design principles that underpin Ir(III) complexes as DNA binders, the biophysical techniques employed to interrogate these systems, and the range of DNA interactions that are demonstrated through both non‐covalent contacts (such as groove binding and intercalation) and covalent bond pathways.

## 3 Biophysical Techniques to Study DNA Binding

Two complementary questions when studying DNA binding of compounds such as iridium(III) complexes are ‘Where does it bind?’ and ‘How strong is the binding?’. The first question centres on structural information, i.e. on the structures and the types of interactions that result in binding. The second question focusses on affinity data, i.e. how strong is the interaction and the answer to this question provides information on concentrations of material that are needed for the interaction to take place. The two questions are complementary, of course, because the molecular structures of the binder and of DNA dictate the interaction mode and thus the nature of the interactions. The interactions between binder and DNA (in combination with effects of desolvation and structural changes) then result in the affinity of the compound for DNA. In the following sections, we will set out a workflow that allows both affinity and structural data to be obtained using techniques that are relatively accessible. The workflow will be presented in the context of Ir(III) complexes interacting with duplex DNA. However, analogous approaches can be applied to other DNA binders and other target structures such as Ir(III) complexes targeting quadruplex DNA structures.

### 3.1 Exploring DNA Binding through Docking Studies

**FIGURE 2:**
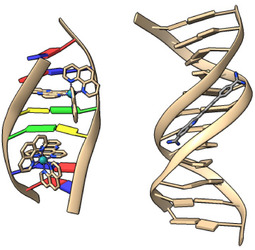
A ruthenium complex binding (left) through intercalation (PDB 4JD8) and small molecule DB1883 binding (right) in the minor groove (PDB 3U0U).

As mentioned above, the four classes of binding modes in which a compound can interact with DNA are 1) intercalation, 2) minor groove binding, 3) major groove binding, and 4) outside electrostatic interactions (intercalation and minor groove binding shown in Figure 2). Outside electrostatic interactions are often accompanied by stacking of the DNA‐binding compounds.

Insight into the feasibility of a compound binding to DNA and the potential DNA‐binding modes can be obtained from molecular docking studies. Molecular docking studies are very accessible and have the advantage that the feasibility of different DNA‐binding modes can be explored before synthesising actual compounds. A variety of docking packages is available, including Intercalate [64] but AutoDock [65] and Autodock Vina [66, 67] have become a popular choice because of their ease of use and the fact that they are open source. Several characteristics should be kept in mind when using AutoDock and AutoDock Vina. Firstly, Vina has been optimised for interactions of small molecules with proteins and not with DNA and only a subset of the elements in the periodic table have been parameterised. Secondly, Vina doesn’t explicitly take electrostatic interactions into account. Finally, like all docking software, the docking targets are typically rigid structures, although some flexible groups can be defined. Despite these characteristics, Vina's performance is easily satisfactory when used with DNA structures, as long as a pre‐formed intercalation gap is present to allow compounds to intercalate if intercalation is a feasible binding mode. We have prepared such a DNA structure with a pre‐formed intercalation gap on the basis of an analysis of DNA structural parameters for intercalation sites [68] while others have complemented docking studies with molecular dynamics simulations to generate DNA structures offering an intercalation gap [57] or used an available structure of DNA with an intercalator and removed the intercalator [58]. The structures of the DNA binders are conveniently obtained from crystal structures, if available, but otherwise straightforward structure minimizations generally suffice because small differences in bond lengths are unlikely to significantly affect the docking results. DFT calculations have also been used to obtain structures suitable for docking [57].

Obviously, care must be taken when interpreting the results. For example, a target structure offering a pre‐formed intercalation site corresponds to the introduction of an energetic bias towards intercalation as the energy cost of forming the intercalation gap is not accounted for. Similarly, calculated binding energies will be affected by the approximations underpinning the docking algorithms, but evaluation of the top binding modes still gives a very good indication of which binding modes are feasible and whether these are likely to be similar in energy or predominantly one or the other. As typical docking runs only involve a single binder and a single target molecule, outside stacking is also not effectively modelled. Finally, for docking studies involving iridium complexes, it should be noted that iridium has not been parameterised. A cost‐effective solution is to temporarily replace the iridium ion with a cobalt ion in the structure of the binder without changing the coordinates of any of the atoms and then carry out the docking studies. The most straightforward approach to replacing iridium with cobalt, without making changes to the complex structure, is to edit the PDB file with the structure of the iridium complex. To replace iridium with cobalt, replace ‘Ir’ with ‘Co’ in the Atom Name and Element Symbol columns of the PDB file using a suitable file editor. Next, the PDBQT file of the ligand can be generated and the docking carried out in the usual way. Before visualising the results, replace instances of Co with Ir in the generated PDBQT files. This solution works well for complexes where 1) the iridium ion is fully surrounded by ligands, i.e. in complexes with DNA there are no direct Ir‐DNA interactions, and 2) there is no significant flexibility in the coordination environment. From a docking point of view, in such cases the iridium centre holds the ligands in place to give the complex its 3D shape but is not directly involved in the interactions with DNA. Because Vina does not use electrostatic interactions, any difference in charges between iridium and cobalt would not affect docking in any case. For complexes where the iridium ion directly interacts with DNA, replacing Ir with Co during docking is not advised.

In addition to evaluation of the feasibility of different DNA‐binding modes, docking studies also provide direct insight into expected binding site sizes; visualisation of the docked structures immediately shows how many basepairs are likely involved in binding with the iridium complex of interest and this then becomes a reasonable expected binding site size which can be compared with binding site sizes as determined from fitting binding models to experimental data.

### 3.2 Biophysical Techniques to Obtain Affinity Data

As mentioned above, affinity data is complementary to structural data. Affinity data can be obtained through a variety of techniques. Here, these techniques will be categorised as either spectroscopic techniques, spectroscopic techniques to obtain thermodynamic data, or other techniques. Throughout the discussion, focus will be upon relatively affordable techniques. However, prior to the discussion of the biophysical techniques, it is essential to consider the aqueous solubility of DNA binders.

Thermodynamic binding models describe equilibria in solution. Because interactions with DNA are most relevant if they take place in water, we assume that experiments are being carried out in predominantly aqueous solutions. It is therefore crucial that aqueous solubility of the iridium(III) complex(es) is evaluated before carrying out experiments to determine affinities. The solubility limit is conveniently evaluated through determination of an extinction coefficient for the iridium complex, followed by determination of the solubility limit by recording a UV–vis spectrum of a filtered or centrifuged saturated solution of the iridium complex. The process of determining an extinction coefficient also provides early quality control because lack of solubility, aggregation and aqueous reactivity may all affect the linearity of the Lambert‐Beer plot.

The extinction coefficient is also useful for the titrations because concentrations of solutions can be determined using UV–vis spectroscopy and the extinction coefficient of the free binder is a parameter in typical binding models for UV–vis titrations. Determining concentrations through the extinction coefficient also means that an the material only needs to be weighed out accurately when determining the extinction coefficient. In practice, this means that less material is needed when preparing solutions for biophysical experiments because the amount of material needed is no longer an accurately weighable amount.

For poorly soluble iridium complexes, non‐volatile co‐solvents such as DMSO can be added to the buffers, although caution is required in the case of organo‐Ir(III) metallocene derivatives such as [(*η* ^5^‐Cp*)Ir(N ^ N)(L)]^0/n+^ (where N ^ N = bidentate ligand; L = monodentate ligand) where L can be labile to coordinating solvents. At high fractions of organic co‐solvent, the DNA double helix may no longer be stable [69, 70], so care should be taken with how much DMSO is added.

### 3.3 Spectroscopic Techniques

#### 3.3.1 UV‐Visible and Luminescence Titrations

Spectroscopic techniques are among the most cost‐effective approaches to study interactions of small molecules and complexes with DNA. Spectroscopic techniques require chromophores, but these are present in the typical structures of iridium complexes which often involve conjugated aromatics as ligands. In addition, many iridium complexes are luminescent, providing further spectroscopic opportunities. As a result, key techniques to study the interactions of iridium complexes with DNA are UV–vis titrations, luminescence titrations, and induced circular dichroism titrations. These direct titrations take advantage of the changing spectroscopic properties of the complexes when the complexes bind to DNA. In cases where the spectroscopy of the iridium complex does not allow direct observation of binding events, indirect spectroscopic techniques such as displacement titrations in which the known intercalator ethidium bromide is displaced by a potential binder of interest can be used.

Direct titrations followed by UV–vis, luminescence, or induced circular dichroism spectroscopy all use a similar approach [71]. First, the iridium complex is dissolved in buffer, the solution is placed in a cuvette, and a spectrum of the complex in the absence of DNA is recorded. Next, the concentration of DNA is increased in a stepwise fashion through the addition of aliquots of a DNA stock solution, and a spectrum is recorded after each addition of DNA. DNA additions are continued until the spectrum of the binder no longer changes (note that spectral features between 200 and 300 nm are generally ignored because this is where DNA absorbs). For data analysis, it is important that a wide range of DNA concentrations is used, ideally at least spanning binding site concentrations from 0.1 × *K*_d_ to 10 × *K*_d_ [72]. Obviously, for the first titration *K*_d_ is unknown, so the precise concentration range is normally determined in an iterative manner. It should be noted that the nature of the DNA stock solution that is added to the solution of the binder affects the subsequent data analysis procedures because addition of a solution of DNA that does not contain the binder will result in dilution of the binder. There are three approaches to deal with the potential dilution of the DNA binder. The first approach is to use a highly concentrated stock solution of DNA so that negligibly small volumes of DNA stock solution need to be added and the concentration of the iridium complex remains the same for all practical purposes. A second approach is to accept that the concentration of the iridium complex decreases during the titration and to take the decreasing concentration into account during data analysis. The third approach is to add the iridium complex to the DNA stock solution at the same concentration as in the sample so that addition of DNA does not dilute the iridium complex.

**FIGURE 3:**
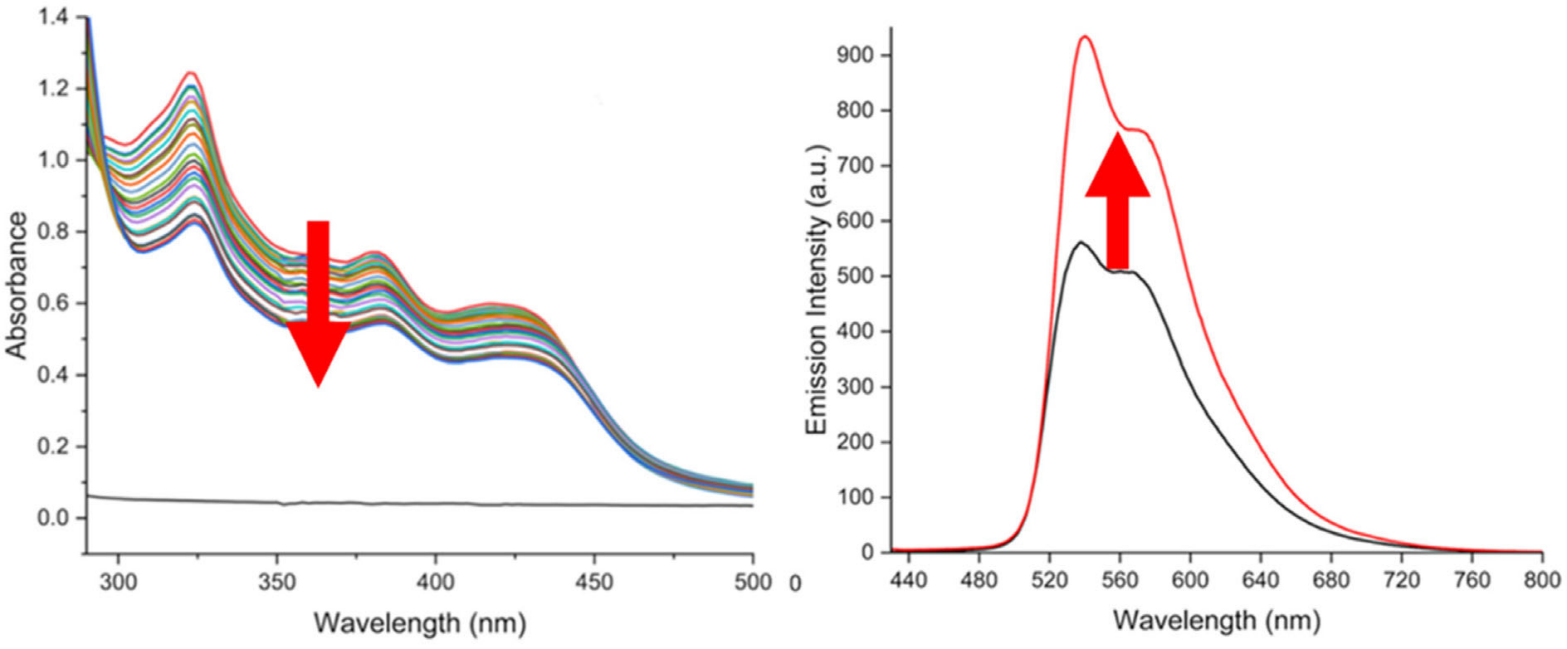
Example changes to the UV‐visible (left) and luminescence (right) spectra for a cationic iridium complex upon addition of DNA. Spectra change as indicated by the arrows. Data taken from SI in Alkhaibari et al., Chem. Eur. J. 2025, 31, e202500290.

Combining the recorded spectra results in an overlay spectrum that shows the effect of increasing concentrations of DNA on the spectroscopic properties of the iridium complex of interest. Typical examples of overlay spectra for the different spectroscopic techniques are shown in Figure 3.

Figure 3 shows examples of changes in spectroscopic properties of a cationic iridium complex as observed by different techniques. UV–vis spectroscopy typically reports on conformational changes and changes in the environment that the Ir(III) complex experiences upon binding to DNA. Luminescence spectroscopy frequently shows either hyper‐ or hypochromic changes in emission intensity, depending on whether binding to DNA reduces quenching of the excited state(s) or provides new quenching pathways. For example, Thomas and co‐workers have observed emission quenching by DNA as a result of the excited state of their iridium complexes photo‐oxidising DNA [73]. It is likely that the redox properties of the excited state play an important role in whether quenching by electron‐transfer can occur. Circular dichroism spectroscopy reports on placing a typically achiral complex in a chiral environment. For example, if an achiral iridium complex is used, the complex itself will not have a CD spectrum. However, when the achiral complex binds to DNA, the transition dipole moment of the iridium complex couples with the transition dipole moments of the DNA bases, which form a chiral environment and thus induce a circular dichroism signal. The induced CD spectrum is therefore a direct indication of an interaction. It is important to note that if the optical isomers (Δ and Λ) of octahedral Ir(III) complexes (e.g. Figure 1) are not resolved, the resultant CD spectra of DNA titrations can be challenging to interpret.

Analysis of the data resulting from spectroscopic titrations requires careful evaluation of data representations and fitting procedures, as demonstrated by an extensive review of fluorescence titrations of the very well‐known DNA binder ethidium bromide [74]. Because DNA is a long molecule and therefore offers multiple binding sites, data analysis methods need to include a parameter to turn the concentration of DNA (which is typically expressed in terms of base pairs) into concentrations of binding sites. Simple 1:1 binding models that take the concentration of DNA expressed in terms of concentration of base pairs as the concentration of available binding sites therefore must not be used. In practice, this means that any analysis in terms of a model that expresses DNA concentration in terms of base pairs but does not explicitly include a binding site size, is highly likely to be incorrect.

Traditionally, a so‐called Scatchard plot was constructed. The parameter *r* is defined as the ratio [binder]_bound_/[DNA base pairs]_total_, and *C*_f_ is the concentration of free binder. Plotting *r*/*C*_f_ as a function of *r* then generates a Scatchard plot [75]. The construction of a Scatchard plot corresponds to linearising the experimental data, but a linear plot is only obtained if the binding constant for all binding sites is constant. More recent data analysis approaches avoid the linearisation of the data and identify a wavelength at which the observed signal, be it absorbance, luminescence, or induced circular dichroism, changes significantly. The signals at this wavelength are then plotted as a function of the concentration of added DNA. In a good titration, a sufficient range of concentrations is used so that the endpoint of titration is visually identifiable as a spectrum that no longer changes at wavelengths above 300 nm (note: DNA absorbs light up to approximately 300 nm) upon increasing the concentration of DNA. Data can also be plotted as a function of the logarithm of the DNA concentration (or the *x*‐axis can be set to be plotted logarithmically) and this should show sigmoidal plots with a mid‐point indicating the *K*_d_ value.

When selecting a binding model to fit to the data, it is important to distinguish between linear fits to linearised data and non‐linear fits to primary data. Historically, data were linearised, such as in a Scatchard plot mentioned above, to allow quantification of the affinity and binding site size directly from a linear fit to the data. This linear fit could be carried out using a ruler if necessary. The drawback to linearising data (to allow a linear fit to be carried out) is that linearisation produces heteroscedastic data, with inconsistent errors on both the *x* and *y* axes. A model should not be fitted to such data [76]. For several decades already, computers allow the iterative fitting of non‐linear models to non‐linearised data and direct fits of binding models to homoscedastic primary data is now commonplace and strongly preferable.

**FIGURE 4:**
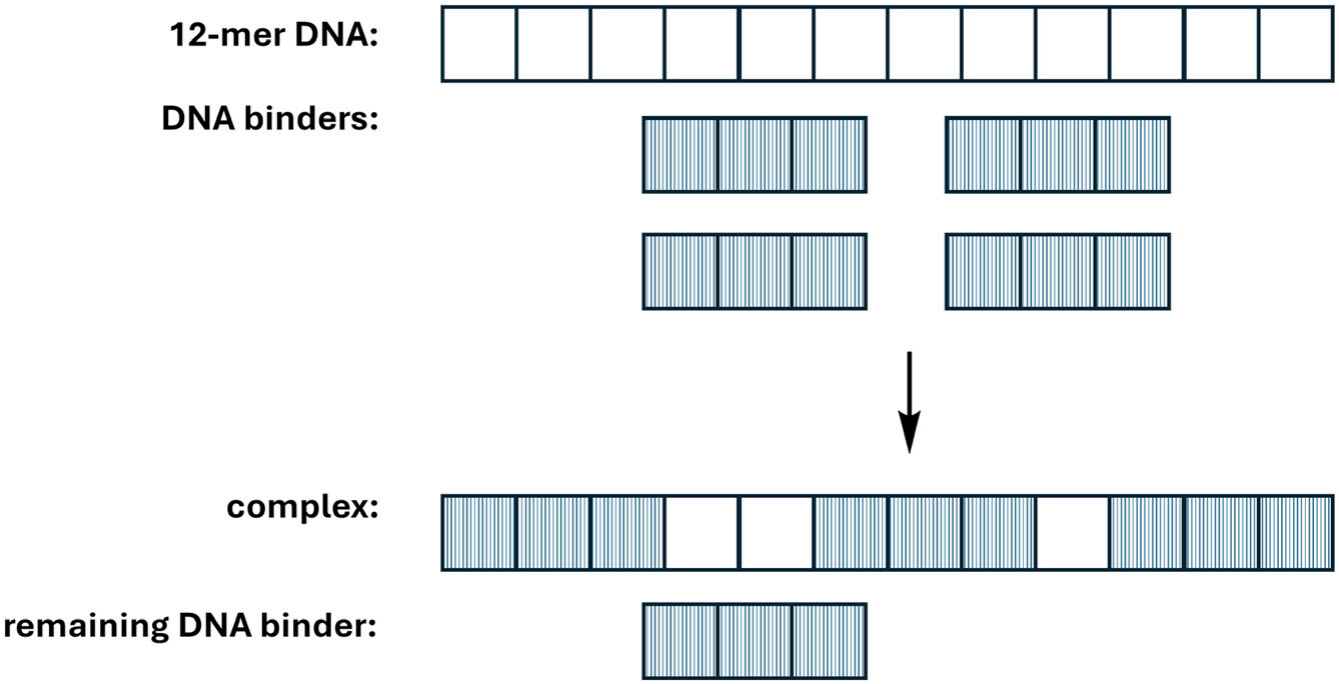
According to the neighbour exclusion model, combining a 12‐mer DNA duplex and a DNA binder with a binding site size of three base pairs does not result in four bound binders per duplex because available base pairs may not be contiguous.

A frequently used model is the McGhee‐Von Hippel model [77] which describes binding of a binder to a homogeneous lattice such as DNA. A key characteristic of the McGhee‐Von Hippel model is the inclusion of the so‐called neighbour‐exclusion effect that takes into account that ‘numerically available binding sites’ may not actually be available because the available base pairs are not contiguous (Figure 4).

The neighbour‐exclusion effect also explains why Scatchard plots for typical experimental data are not linear, even if the binding constant may be expected to remain the same for all binding events. The further inclusion of cooperative or anti‐cooperative binding in the McGhee‐Von Hippel model has been proposed as well but often suffers from lack of accuracy in quantifying the cooperativity parameters [78]. Where non‐homogeneous DNA such as fish sperm DNA or calf thymus DNA is used in a titration, the risk in over‐interpreting curvature in Scatchard plots in terms of cooperativity is even higher, because curvature is actually the result of the natural variation in affinity of the binder for the different sequences present on the DNA.

Maybe counterintuitively, the use of simple binding models provides the safety of not over‐interpreting intricacies of the data in terms of cooperativity. For this reason, the multiple independent binding sites model as described by Crothers, which simply describes DNA as a sequence of binding sites, remains popular. Our binding model, which takes the decreasing concentration of the DNA binder during the titration into account [79], is a version of this model.

An additional advantage of modern curve‐fitting software is that multiple data sets can be analysed at the same time in a global fitting approach. This means that data at several wavelengths, at several concentrations of the iridium complex and even from different spectroscopic methods can be combined and analysed in terms of a single set of binding parameters *K*_a_ and *n*. The advantages of global fitting are that one either gets a better quantification of the parameter values or a first indication that the binding model cannot reproduce the data across all wavelengths and combinations of concentrations. In such cases, alternative binding models should be considered. Data from additional techniques beyond spectroscopy then become beneficial as well.

The extension of using data at multiple wavelengths, and potentially from several spectroscopic techniques, is the use of singular value decomposition of the spectroscopic data over the full range of absorbance as visible in the overlay spectrum. This approach has, to our knowledge, not yet been used for the analysis of spectroscopic data for iridium complexes interacting with DNA.

Once a model has been fit to the titration data, several checks should be carried out on the parameters. First of all, the error margins and potential parameter covariance should be evaluated, in particular for binding models involving several binding modes. To reiterate, the simplest reasonable binding model that reproduces the data well should be used to avoid over‐fitting the data. The fitted stoichiometry should reasonably resemble the expected binding site size from the molecular docking. Reasonable resemblance includes the potential effects of the binder on adjacent base pairs. For example, a typical binding site size for intercalators is three base pairs because intercalation between two base pairs also affects the adjacent base step. If binding sites are significantly larger than expected on the basis of the size of the binder, this may indicate selectivity for a sequence of structure. On the other hand, fitted binding site sizes may be much smaller than the anticipated binding site size. In our experience, this often indicates the presence of multiple types of binding sites. Typically, the types of binding sites in this scenario are a higher affinity binding site with a binding site size larger than one base pairs in combination with outside electrostatically driven stacking of DNA binders as a secondary binding site. In terms of data analysis, a significant problem with this scenario is that, in titrations where DNA is added to the iridium complex, the earliest datapoints correspond to an excess of the binder relative to the DNA. As result, all binding sites on DNA are occupied at the start of the titration and further addition of DNA then results in binders moving from secondary binding sites to primary binding sites and not to free binders binding to DNA. In the worst case, this scenario is accompanied by precipitation of the DNA–binder complex as a result of the cationic iridium complex cancelling out the negative charges of the DNA. Such precipitation may be visible as a slight cloudiness to the solution. In the recorded UV–vis spectra, precipitation may also show up as a sharp drop in absorbance at the λ_max_ of the iridium complex upon the addition of the first few aliquots of DNA and a small increase in measured absorbance because of light scattering at wavelengths where the iridium complex doesn’t absorb light. Sometimes, however, the only warning sign suggesting the presence of two binding modes is the unexpected stoichiometry and our previous studies have shown that a simple model may accidentally reproduce the experimental data in a satisfactory manner, but that the resulting binding parameters should then be interpreted with care (see supporting information within reference [79]).

As alternatives to direct titrations, indirect displacement titrations of well‐known DNA binders such as ethidium bromide ensure that a suitable chromophore is present. Such displacement titrations are often used because of the structural information they provide [80].

Finally, an extension of luminescence spectroscopy titrations involve the measurement of luminescence emission lifetimes. For example, time‐resolved emission measurements can quantify the increased lifetimes of the excited state of iridium(III) complexes when bound to DNA [81], while others have taken advantage of time‐resolved emission spectroscopy to separate the long lifetime luminescence of an iridium complex from background fluorescence [82].

#### 3.3.2 Isothermal Titration Calorimetry

Of the techniques that don’t rely on spectroscopy, isothermal titration calorimetry (ITC) is one of the most versatile because it measures the intrinsic heat effects associated with the interaction between the iridium complex and DNA. ITC is the gold standard for studying molecular interactions in solution, not only because it provides direct access to thermodynamic parameters but also because ITC is extremely unlikely to produce data resembling an interaction without the interaction really taking place. ITC is also reasonably well accessible, and titrations are fully automated as standard.

**FIGURE 5:**
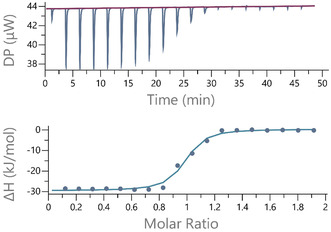
ITC records heat flow DP as a function of time. Heat flow spikes following injection of a binder into a DNA solution are integrated to yield molar heat effects.

Briefly, in a typical ITC experiment, the iridium complex and the DNA are dissolved in a matched buffer. Matching the buffer is important because ITC is a very sensitive technique that will also measure the mixing heat effects of unmatched buffers. The DNA solution is placed in the sample cell, and the solution of the iridium complex is placed in an automated titration syringe and titrated into the DNA solution. Heat flow to the sample cell is recorded as a function of time. Integration of the heat flow and division of the integrated heat per injection by the number of moles of iridium complex injected then results in molar heat effects which can be plotted as a function of molar ratio. Figure 5 shows general example data for an ITC experiment involving an exothermic 1:1 binding event.

Ideal titration curves show sigmoidal behaviour. Whether a sigmoidal curve is obtained depends on the so‐called *c*‐value, which is the product of the concentration of binding sites in the calorimeter sample cell and the affinity of the iridium complex for those binding sites *K*_a_. If the c‐value is >5, then the sigmoidal binding curves allow direct quantification of the thermodynamic binding parameters. Experiments at lower *c*‐values are also possible but only provide acceptable quantification of the thermodynamic parameters under a very strict set of conditions [83, 84]. A further requirement for ITC experiments is that the concentrations used in the experiment are such that measurable heat signals are observed. In practice, this means that concentrations of binding sites in the calorimeter sample cell typically range from 5 to 500 μM. Because of the relative volumes of the sample cell and the injection syringe, the concentration of the iridium complex in the injection syringe needs to be approximately ten times higher than the concentration of binding sites in the sample cell to get a titration where the mid‐point of the titration (1:1 binding sites:iridium complex) lies midway the titration.

In principle, titrations can also be carried out in which DNA is titrated into a solution of the iridium complex. However, a key benefit of ITC experiments is that the usual titration approach results in a titration in which binding to stronger binding sites dominates at the start of the titration with binding to weaker binding being visible later in the titration. This is complementary to typical spectroscopic titrations where the excess of binder to DNA at the start of a titration means that all binding sites are filled at the start of the titration, followed by migration from weaker to stronger binding sites as the titration proceeds (vide supra).

A further advantage of ITC is that the technique provides excellent insight into aggregation of the interacting species, which may not be noted in spectroscopic titrations. For example, the aggregation of some water‐soluble cationic iridium complexes in aqueous solution has been shown to be sensitive to counteranion concentration [81]. Because self‐aggregation of iridium complexes is in competition with DNA binding, it needs to be addressed either by changing the experimental conditions or during data analysis.

The fact that ITC is sensitive to all interactions that are accompanied by heat effects is an advantage but also means that data analysis needs to describe all interactions that are measured. Models that include many optimisable parameters, however, are sensitive to parameters covariance. It is therefore useful to reduce the complexity of the equilibrium system and resulting heat effects as much as possible. Reducing data complexity starts with using matched buffers to remove any heat effects of buffer mixing but also includes avoiding aggregation of the DNA binder if possible. For example, in our own studies, prevention of Ir(III) complex aggregation was achieved by changing buffer conditions [81].

In ideal cases, removing complexity results in sigmoidal data that can be analysed using standard software as provided by the instrument manufacturer. Often, however, data are more complicated and require custom data analysis models. Possibly the best model for the analysis of ITC data for binding to homogeneous DNA sequences where bound DNA binders interact with each other is the model developed by Lincoln and co‐workers [85]. If there are several types of binding sites or if the DNA binder also aggregates in solution, our data analysis software I2CITC [86, 87, 88] can be used.

A significant advantage of ITC in studying interactions with DNA is that different types of binding sites are often more obvious in titration calorimetry than in spectrometric titrations. For example, we have used ITC to demonstrate two different binding modes of an Ir(III) complex interacting with DNA [81].

Analysis of these data in terms of a binding model involving two types of binding sites provided equilibrium constants and binding site sizes for both types of binding sites. For complex equilibrium models, evaluation of error margins on parameters and parameter covariance should also be carried out [88]. As before, the resulting binding parameters *K* and *n* should be evaluated in terms of their physical meaning. If binding sites are significantly bigger than expected, this may indicate selectivity for a sequence or structure and binding site sizes around 1 base pair likely correspond to outside stacking of cationic binders directed by the anionic DNA polymer.

The further thermodynamic parameters obtained from ITC experiments can be interpreted in terms of a binding mode through an empirical correlation between Δ*H* and DNA‐binding mode [89]. Moreover, ITC experiments can also be used to study binding‐linked (de)protonation events [90]. Finally, heat capacity changes can be determined from ITC experiments at different temperatures, and these can be interpreted in terms of an estimate of surface accessible surface burial through an empirical correlation [91].

#### 3.3.3 Spectroscopic Techniques to Obtain Thermodynamic Data and Other Techniques to Explore Binding

In addition to monitoring the effects of DNA binding on the spectroscopic properties of the iridium complexes, it is also possible to use spectroscopy to study the effect of added iridium complexes on the stability of the DNA double helix or G‐quadruplex fold. Typically, this involves recording UV–vis or circular dichroism spectra of a DNA solution while the temperature is slowly raised through the so‐called melting temperature of the DNA (*T*_m_) at which the double helix dissociates into two separate single strands or the quadruplex unfolds. For systems where standard spectroscopic methods cannot be used to study DNA melting, FRET (Förster resonance energy transfer) melting experiments can be used. In these experiments, each strand forming the double helix (or appropriate sites in a quadruplex‐forming sequence) is modified with a fluorophore where the two fluorophores form a so‐called FRET pair. Duplex formation or quadruplex folding places the fluorophores together so that FRET is observed. Above *T*_m_, the fluorophores are separated and FRET efficiency decreases. This approach has been used, for example, to demonstrate the stabilising effect of an iridium complex binding to a quadruplex DNA [82]. Analysis of the resulting melting profiles in terms of the Van‘t Hoff equation allows quantification of the thermodynamics of DNA duplex formation.

These melting profiles depend on DNA concentration, salt concentration and added DNA binders. When data are recorded in the absence and presence of added DNA binder, but under otherwise identical conditions, a duplex‐stabilising effect of the DNA binders is typically observed. The stabilising effect is expressed in terms of an increase of melting temperature Δ*T*_m_ and a positive Δ*T*_m_ is an indication of binding to duplex DNA.

In addition to the techniques discussed above, new biophysical techniques are currently being commercialised. It is anticipated that new techniques such as switchSENSE [92, 93, 94] and grating‐coupled interferometry [95] will also be used to study the affinity of Ir(III) complexes (and related species) for DNA in the near future.

### 3.4 Structural Information on DNA Binding

To understand by which binding mode(s) a compound interacts with DNA, specialist structural techniques such as NMR spectroscopy and X‐ray diffraction of crystal structures can be used to obtain experimental data leading to structural models with atomic resolution. These techniques are typically used through collaboration with specialists and fall outside the scope of this review; the reader is referred to references [96, 97, 98] for further detail.

Structural information on DNA binding modes can also be obtained from interpretation of linear dichroism (LD) spectra [99, 100, 101]. Like the structural techniques mentioned above, linear dichroism is a technique that is typically used in collaboration with specialists. Briefly, long strands of DNA are aligned through shear. LD is sensitive to the orientation of the transition dipole moments of the chromophores relative to the aligned DNA helix axis. Typically, intercalation results in a negative LD signal for the ligand's absorption band because the transition dipole is perpendicular to the DNA helical axis while groove binding produces a positive LD signal for the ligand's absorption band because the ligand orients along the groove, which is roughly parallel to the helix axis. While LD has not, to the best of our knowledge, been used to study Ir(III) complexes, data for cationic ruthenium complexes have been reported (e.g. Ref. [102, 103]).

More readily available techniques to obtain structural insight are circular dichroism spectroscopy, viscometry, and molecular docking. Induced circular dichroism spectra are useful in titrations to quantify affinity for DNA (vide supra). Such titrations also yield the induced circular dichroism spectra, and these spectra can be qualitatively interpreted in terms of binding mode using empirical relationships. These relationships include the observation that larger induced circular dichroism signals suggest groove binding. For compounds binding as a dimer in the groove, a bisignate signal is often observed. The sign of the induced circular dichroism signal for intercalators depends on the orientation of the transition dipole moment relative to the pseudo‐dyad axis (which is similar to, but not the same as, the DNA helical axis) [71, 101].

**FIGURE 6:**
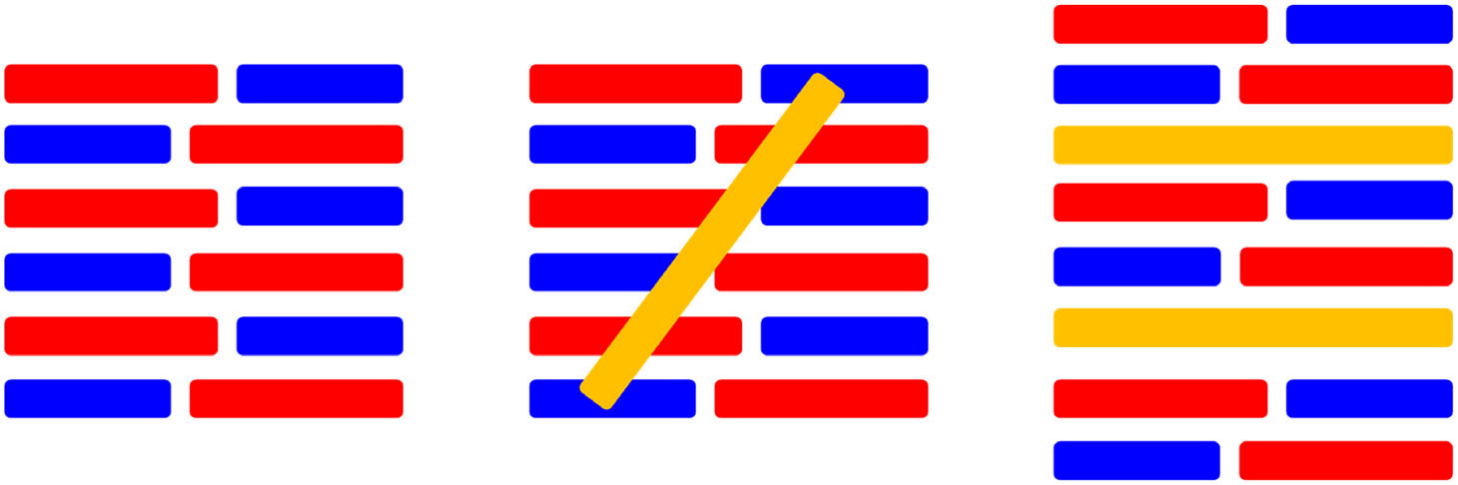
Cartoon representation of duplex DNA length, for groove binding and for intercalation binding modes.

A particularly cost‐effective approach to studying the binding mode of DNA binders is offered by viscosity measurements [104]. Viscosity measurements use the fact that intercalation of a DNA binder requires the formation of an intercalation gap, which moves adjacent base pairs away from each other to create space for the intercalator. This process results in an increase in the length of the DNA–binder complex. In contrast, groove binding does not result in an increase in the length of the complex (Figure 6).

As a result, binding of an intercalator to a solution of highly polymeric DNA results in an increase in relative solution viscosity (*η*/*η*_0_)^1/3^ while groove binding does not. Viscometry has been used to study the DNA binding mode of several Ir(III) complexes.

Competition experiments can also provide insight into binding modes. In these experiments, a known intercalator such as ethidium bromide and a known groove binder such as DAPI (4′,6‐diamidino‐2‐phenylindole) are used and pre‐formed complexes of these compounds with DNA are titrated with the DNA binder of interest. If the binder displaces ethidium bromide, then it is likely an intercalator. If the binder displaces DAPI, it is likely a minor groove binder. Displacement titrations involving just ethidium bromide are a popular choice in studies of Ir(III) complexes interacting with DNA, but displacement titrations involving multiple DNA binders such as ethidium bromide and DAPI have been carried out as well [105].

With the exception of the techniques providing atomic resolution structures, none of the techniques above provide a definitive to answer to the binding‐mode question, and it is therefore best to obtain data from several techniques and combine this with the results from molecular docking studies to gains the best possible insight into the binding mode.

## 4 Background on Photoluminescent Ir(III) Complexes

The use of luminescence spectroscopy is a powerful tool in the analysis of DNA interactions with molecular probes. The purpose of this section is to provide a brief description of the photoluminescent properties, and consequences for design, of Ir(III) complexes that are useful in DNA binding studies. As noted earlier, half‐sandwich metallocene Ir(III) species generally have poor intrinsic photophysical attributes due to the moderately weak ligand field and low‐lying metal‐centred d states that provide effective routes for non‐radiative deactivation [106, 107]. In these cases, emissive character is imparted by considering either highly conjugated ligands which can facilitate population of emissive ligand‐centred triplet states [108] or ligand conjugation with aromatic fluorescent labels (e.g. BODIPY) which are then useful in live cell bioimaging experiments [109].

Despite Ir(III) polypyridines being attractive in DNA studies, they are far less reported in the literature compared to their cyclometalated cousins. Octahedral polypyridine Ir(III) species appear to be more challenging to synthesise and purify in good yield, often due to competing orthometalated byproducts (especially in the case of unsubstituted bipyridines). However, reports have shown that using, for example, 4,4’‐dimethyl‐2,2’‐bipyridine (dmbipy) can yield complexes [Ir(dmbipy)_2_(L)]^n+^ which demonstrate phosphorescent (typically microsecond domain lifetimes and easily discriminated from shorter lived fluorescent species) emission ca. 500 nm; [110] homoleptic [Ir(phen)_3_]^3+^ (phen = 1,10‐phenanthroline) has reported emission at 455 nm [111]. In such complexes, the emission band shapes are often characterised by strong vibronic coupling and microsecond scale lifetimes, suggesting a ligand‐centred triplet excited state (the high spin orbit coupling constant associated with the heavy Ir atom facilitates efficient triplet population on the ligands) rather than charge transfer (CT) triplets that are typically more bathochromically shifted.

The most common class of photoluminescent organometallic Ir(III) complex are those that encompass two or three anionic cyclometalating ligands (C ^ N) as part of an octahedral coordination sphere, giving general formulae of [Ir(C ^ N)_2_(L)]^n±^ (where L = bidentate ligand) or [Ir(C ^ N)_3_], respectively. The latter [Ir(C ^ N)_3_] species are typically charge neutral and have been successfully applied to electroluminescent devices; in contrast, because they offer restricted solubility in aqueous media (unless ligands are suitably adapted) they appear far less prominently in DNA studies. An overall cationic complex, however, offers clear advantages since counter ion choice can be optimised, and favourable electrostatic interactions with negatively charged DNA can be promoted. Therefore, heteroleptic bis‐cyclometalated complexes, [Ir(C ^ N)_2_(L)]^+^, are more attractive (for example in the case of [Ir(C ^ N)_2_(bipy)]^+^, where the ancillary bipy is neutral). Developments in the rational synthesis [112] and purification of cyclometalated species can allow access to heteroleptic species [Ir(C ^ N)(C ^ N’)(L)]^n±^ where all three ligands are distinct, giving exceptional control over the (photo)physical properties of the complex [113]. While they often possess a lower magnitude of charge compared to the related Ru(II)‐polypyridines and, indeed, Ir(III) polypyridines, these general observations become more nuanced if one considers the use of cationic ligands.

**FIGURE 7:**
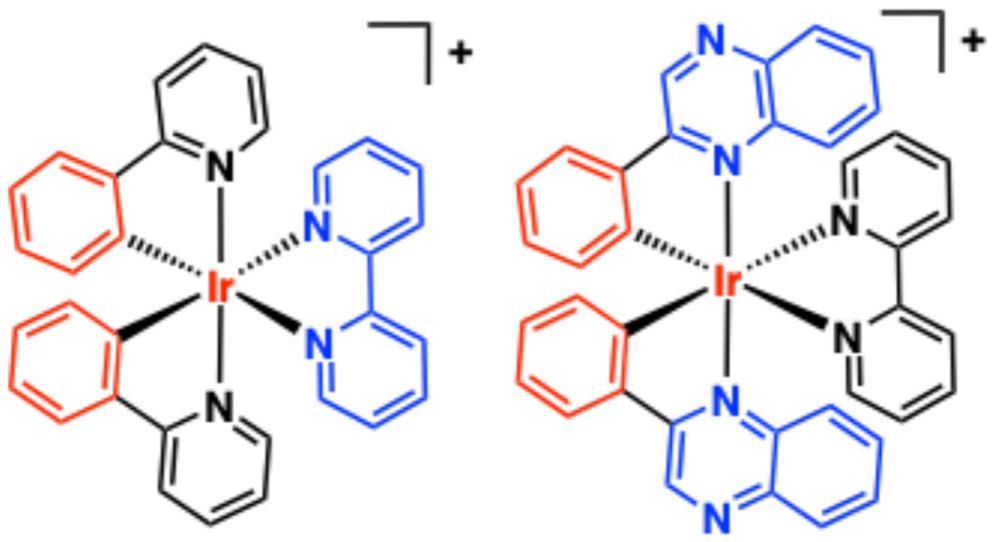
Representation of predominant HOMO (red) and LUMO (blue) location that dictate photoluminescence in two closely related cationic Ir(III) architectures.

Critically, the choice and combination of ligands in cyclometalated Ir(III) complexes dictates the photoluminescent properties, providing opportunity to tune desirable attributes. This area of research has been extensively studied for >20 years [114]. As a benchmark, the most common C ^ N ligands are derivatives of 2‐phenylpyridine (ppy); [Ir(ppy)_2_(bipy]^+^ is phosphorescent at 602 nm (MeCN) due to an admixture of ^3^MLCT/^3^LLCT states, which can be long‐lived (up to microseconds) and efficient (quantum yields 25–40% and not uncommon). The locale of the important HOMOs and LUMOs can be dictated through choice of ligand(s) (Figure 7). For example, in [Ir(ppy)_2_(bipy]^+^, the HOMO encompasses Ir 5d and phenyl MOs, while the LUMO is mainly localised on the bipy. In comparison, the green emitting [Ir(ppy)_2_(acac)] has similar HOMO character, but the LUMO is localised on the pyridine ring of ppy, i.e. the cyclometalating ligand, strongly dictates emission character (from ^3^MLCT/^3^LC states). This is also true when more conjugated cyclometalating ligands such as 2‐phenylquinoxaline (2‐pqx) are used instead of ppy; for 2‐pqx, the important LUMO is localised on the quinoxaline portion of the C ^ N ligand [115, 116, 117, 118], and provides an entry point for rational tuning of emission to the red and near‐IR region [119, 120]. These considerations are critical in the rational design of an Ir(III) DNA binder where the modulation photophysical parameters is sought.

## 5 DNA Binding Behaviour of [Ir(bipy)_2_(dppz)]^3+^ Versus [Ru(bipy)_2_(dppz)]^2+^

The relevance of polycationic Ir(III) polypyridines becomes notable when considering benchmark metallointercalators of DNA. Ru(II)‐polypyridine complexes have been extensively studied and reviewed with respect to their DNA binding behaviour [121]. The mixed ligand species [Ru(N ^ N)_2_(dppz)]^2+^ (where N ^ N = diimine such as 2,2’‐bipyridine or 1,10‐phenanthroline; dppz = dipyrido‐[3,2‐a:2’,3’‐c]‐phenazine) is perhaps the most famous metal complex DNA intercalator of all, including for the selective detection of non Watson‐Crick base pair mismatches [122]. The dicationic charge of [Ru(N ^ N)_2_(dppz)]^2+^ promotes favourable electrostatic attraction with anionic DNA, and the extended planarity and hydrophobicity of the dppz ligand facilitates intercalation with DNA. Numerous studies, especially those by Barton and co‐workers [123, 124], have described the dual functionality of this complex, and variants, as a luminescent light‐switch system for DNA; once bound to DNA the emission intensity is ‘switched on’ primarily because the phenazine localised excited state is shielded from quenching solvent. Indeed, the photophysical origin of the light switch effect of these complexes with DNA continues to be studied three decades later [125, 126]. Therefore, in the initial discussion of Ir(III) complexes as candidates for DNA binding studies, it is contextually important to compare analogous Ru(II) and Ir(III) dppz complexes relevant to DNA binding.

**FIGURE 8:**
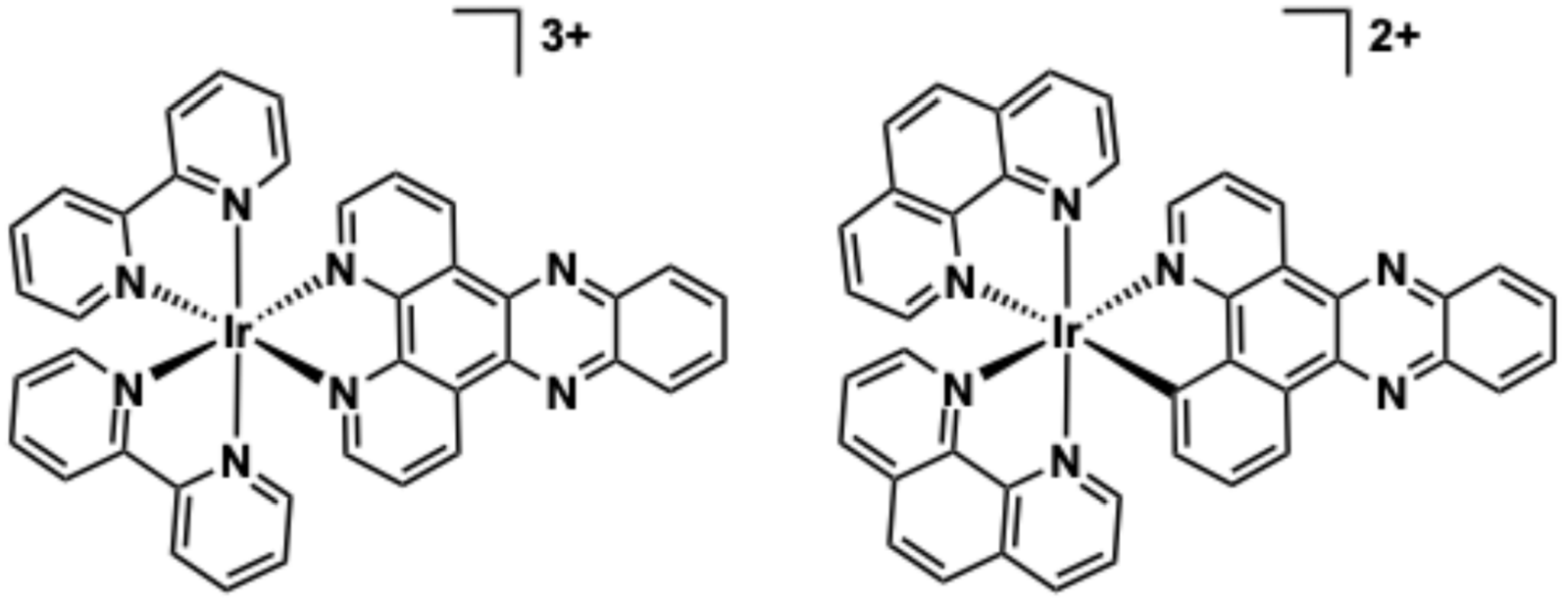
Molecular structures of complexes **1**–**2**.

While Ir(III) complexes share the same low spin d^6^ electronic configuration as Ru(II) and possess (pseudo)octahedral geometry and kinetic inertness which can favour applications in a biological context, it was only in 2015 that Thomas and co‐workers reported the water soluble complex, [Ir(bipy)_2_(dppz)]^3+^ (**1**), which is isostructural (Figure 8) with the archetypal DNA binder [Ru(bipy)_2_(dppz)]^2+^ [127]. The optical and electronic properties of [Ir(bipy)_2_(dppz)]^3+^ are different to the Ru(II) benchmark. Firstly, **1** is emissive in water (*λ*_em_ = 479 nm), with a vibronically structured band profile which is attributed to an intraligand excited state localised on dppz. Upon addition of ct‐DNA the emission is gradually quenched (proposed to occur via a redox process involving nucleobase sites) and therefore does not show the same “switch on” ^3^MLCT state behaviour as [Ru(bipy)_2_(dppz)]^2+^. Through these measurements and viscosity enhancement determinations, **1** was shown to intercalate into duplex DNA, with an affinity (using the McGhee‐von Hippel model) of 1.8 × 10^6^ M^−1^, which is comparable to the Ru(II) analogue. Complex **2**, [Ir(phen)_2_(bppz)]^2+^, is structurally similar, but bipy is replaced by 1,10‐phenanthroline (phen) and the phenazine ligand (bppz = benzo[*a*]pyrido[2,3‐*c*]phenazine) is now cyclometalated (i.e. anionic) resulting in a dicationic analogue. **2** also binds to DNA in an intercalative manner with a similar affinity (2.7 × 10^6^ M^−1^) that is driven by hydrophobic effects as deduced through steady state luminescence titrations; a broad luminescence profile (*λ*_em_ = 522 nm) may suggest a stronger MLCT component (although supporting emission lifetime data are not described), and addition of DNA again quenches the emission; no light switch effect was observed. The study of compounds **1** and **2** shows that while the DNA binding behaviour is comparable, the photoluminescent output of Ir(III) complexes vary substantially from analogous Ru(II) systems and must be considered in the design of Ir(III) probes for DNA.

## 6 DNA Binding Behaviour in Other Ir(III) Polypyridines

**FIGURE 9:**
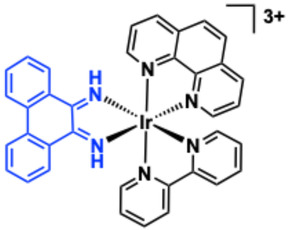
Structure of complex **3** (phi ligand is highlighted in blue).

Barton and co‐workers engineered a heteroleptic Ir(III) complex **3** (Figure 9) [128] that incorporates phenanthrene‐9,10‐diimine (phi‐well known in related Rh(III)‐based metallointercalators [129, 130, 131]) to study DNA interactions. Complex **3** was resolved into its Δ and Λ enantiomers using chiral chromatography, with configurations confirmed by CD spectroscopy. This enantiomeric resolution enabled precise investigation of chirality‐dependent DNA binding. The phi ligand of complex **3** promotes intercalation into DNA, as evidenced by UV–vis titrations showing 35% hypochromism and a 10 nm red shift. A high binding affinity (*K*_*b*_ ≈ 1.1 × 10^6^ M^−1^) was determined by electrochemical titration at DNA‐modified electrodes [132]. Cyclic voltammetry revealed a reversible one‐electron phi‐centred reduction in solution (−0.025 V vs Ag/AgCl), which converted to a concerted two‐electron process upon DNA binding. Electron paramagnetic resonance (EPR) spectroscopy revealed a phi‐based radical in solution and a DNA‐induced diradical species.

**FIGURE 10:**
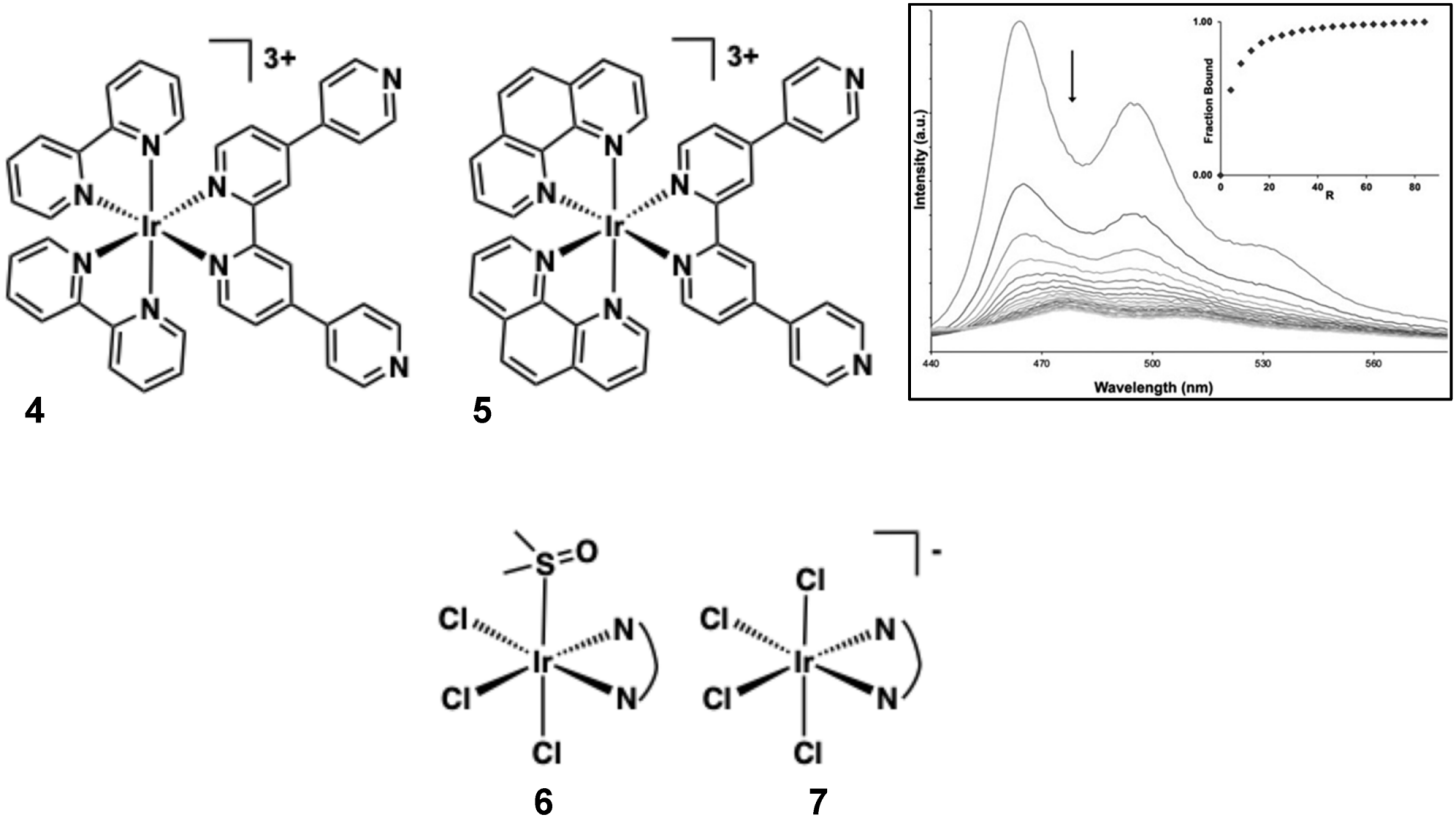
Structures of complexes **4–7**. Shown inset: changes in the luminescence of a buffered aqueous 100 μM solution of **5** on addition of ct‐DNA, up to excess (with binding curve constructed from these data, where R is the binding ratio). Reproduced with permission [73]. Copyright 2024, Royal Society of Chemistry.

Thomas and co‐workers designed two tricationic polypyridyl Ir(III) complexes, **4** and **5** (Figure 10), incorporating the extended quaterpyridyl (qtpy) ligand to enhance DNA interactions [73]. As their chloride salts, both complexes displayed good water solubility and showed strong luminescence quenching upon addition of duplex DNA. Luminescence titrations fitted to the McGhee‐von Hippel model revealed high binding affinities (*K*_*b*_ = 3.7 × 10^6^ M^−1^ for **4** and 1.5 × 10^6^ M^−1^ for **5**), significantly exceeding those of their isostructural Ru(II) analogues [133, 134, 135]. CD spectra showed no disruption of duplex helicity, which supported an assignment of groove binding. In addition, molecular dynamics simulations suggested a predominant minor groove binding mode, with the qtpy ligand aligning parallel to the groove. Complex **5** also exhibited a secondary binding motif involving facial insertion of a phen ligand. Photocleavage experiments showed that complex **4** functions as a light‐activated photonuclease, inducing concentration‐dependent strand breaks [136].

Sheldrick and co‐workers reported cytotoxic trichloroiridium(III) species (structure **6**) of the form *fac*‐[IrCl_3_(DMSO)(N ^ N)] (where N ^ N = bipy, phen, dpq, dppz, dppn) [137]. While these compounds lack helpful photophysical attributes (in fact these complexes are prone to fac/mer photoisomerism), they are nonetheless biologically potent. DNA interactions were studied using UV–vis spectroscopy and thermal denaturation experiments and showed that only the dppz variant showed a weak DNA intercalation; an absence of DNA interactions were noted for all other complexes in the series. Surprisingly, related anionic complexes (structure **7**) of the formulation [IrCl_4_(N ^ N)]^‐^ (where N ^ N = 2,2′‐bi‐imidazole or 2‐(2′‐pyridyl)benzimidazole) have been proposed to demonstrate binding interactions with a number of biological macromolecules (e.g. human serum albumin) including ct‐DNA. UV–vis DNA titrations and thermal denaturation suggest an interaction with DNA (∼10^4^ M^−1^) that may be due to groove binding. H‐bonding interactions between the coordinated ligand and DNA base pairs may help to overcome any inherent electrostatic repulsion [138].

## 7 Cyclometalated Ir(III) Complexes that Bind with DNA

**FIGURE 11:**
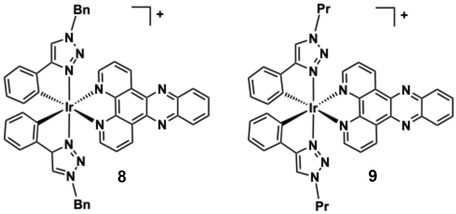
Structures of complexes **8**–**9**.

Two structurally related organometallic Ir(III) complexes, **8** and **9**, both incorporate a dppz ancillary ligand, but differ in their phenyl triazole cyclometalating ligands (Figure 11) (*N*‐substituted with either benzyl or propyl groups) [139]. UV–vis titrations of both complexes showed hypochromicity upon DNA addition, indicating interaction with the DNA helix [127]. Both complexes exhibited enhanced luminescence upon binding to duplex DNA, consistent with an intercalative binding mode and suggestive of emission from a Ir → dppz ^3^MLCT state. This behaviour demonstrates a prospective light switch effect, where emission was enhanced due to shielding of the dppz unit from quenching in aqueous media [140]. The DNA binding affinities (2.38 × 10^4^ M^−1^ for **9** and 5.37 × 10^4^ M^−1^ for **9**) were determined using the MVH model, and the lower affinity of **8** is likely due to the pronounced steric hindrance of the more bulky benzyl group on the C ^ N ligands.

**FIGURE 12:**
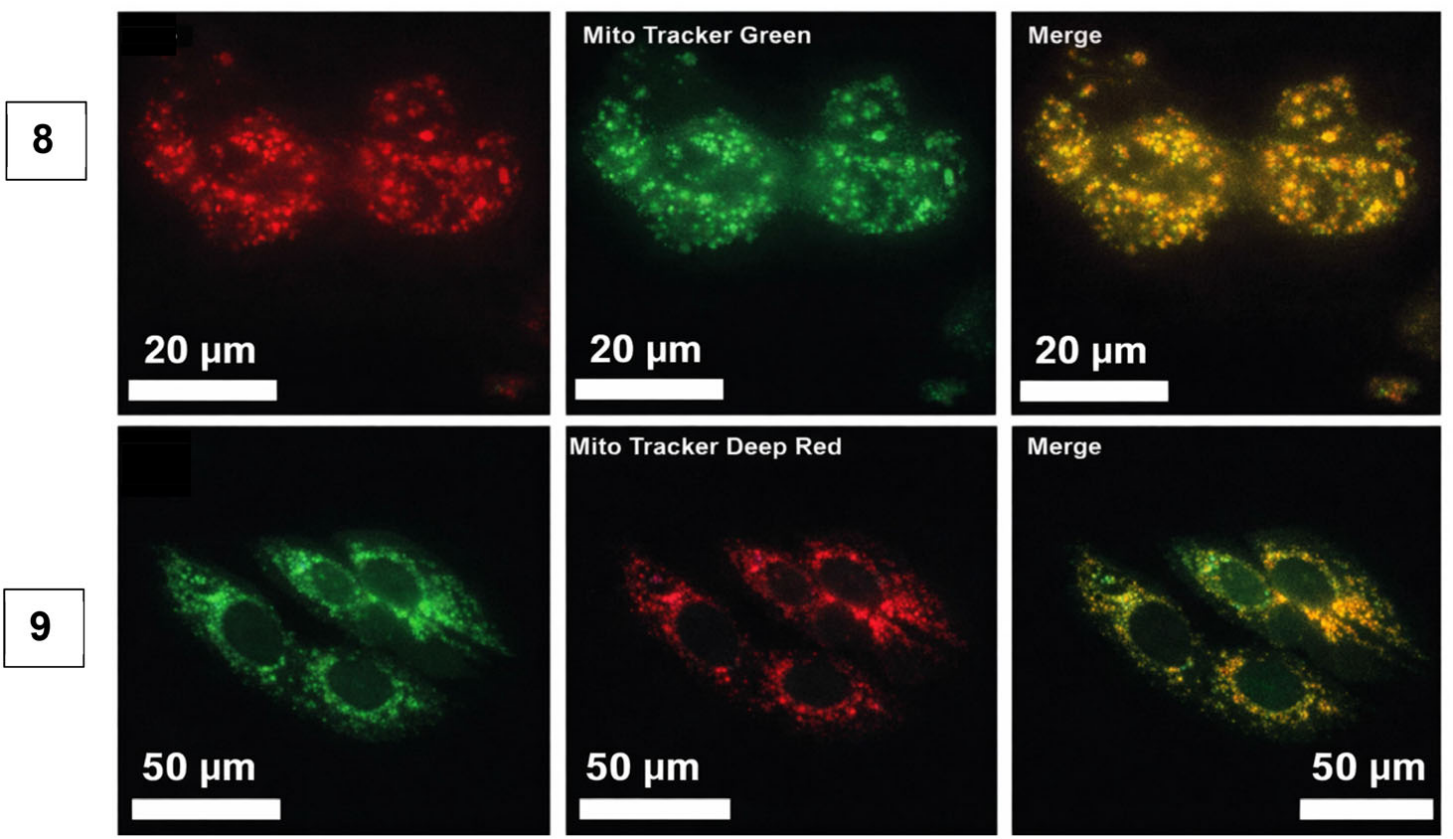
Co‐localisation studies of complex **8** with Mitotracker green (top) and complex **9** with Mitotracker deep red (bottom) in A2780 cells using fluorescence microscopy. Reproduced and adapted with permission. Copyright 2018, Royal Society of Chemistry.

The photoluminescence character of **8** and **9** was further exploited in confocal fluorescence studies of A2780 cells. Co‐localisation studies show they clearly accumulate in mitochondria (but not the nucleus), and this study is an example of the viability of organometallic complexes for targeted fluorescence imaging of live cells (Figure 12).

**FIGURE 13:**
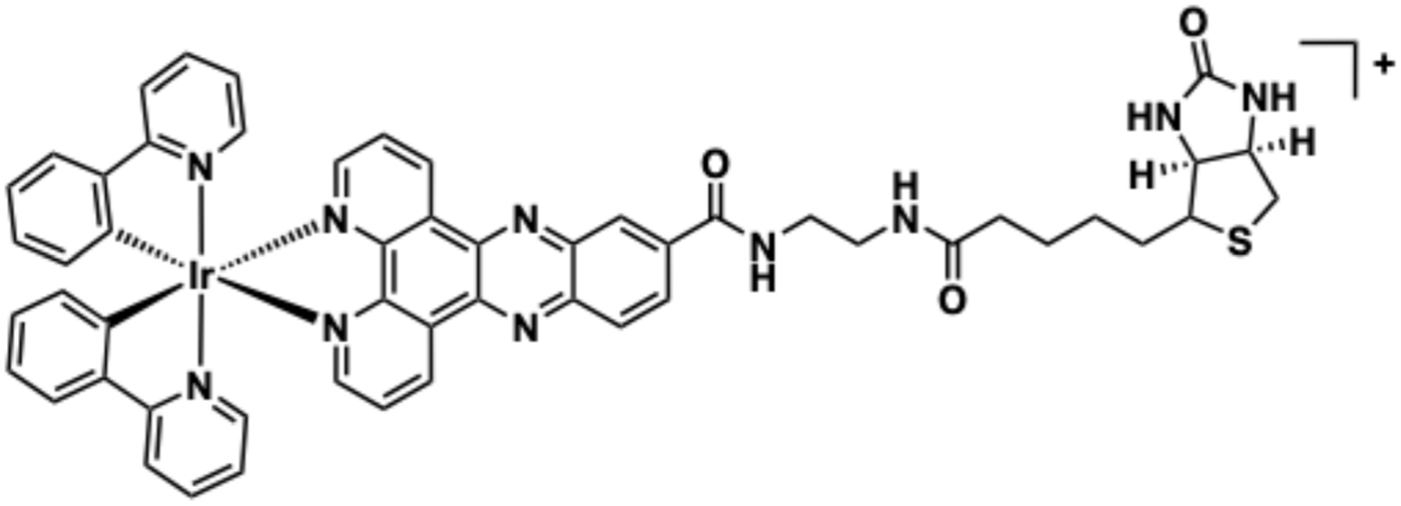
Structure of complex **9**.

Lo and co‐workers reported a series of functional luminescent [Ir(ppy)_2_(L)]^+^ complexes (where L = functionalised diimine ligand) as DNA intercalators and avidin probes [141]. Among these, both [Ir(ppy)_2_(dppz)]^+^ and [Ir(ppy)_2_(dppn)]^+^ exhibited intercalative binding to ct‐DNA with *K*_*b*_ ≈ 2.0 × 10^4^ M^−1^ and 7.8 × 10^4^ M^−1^ respectively (from Bard/Thorp model), as indicated by hypo‐ and bathochromic shifts in UV–vis titrations, as noted in Cu(II) complexes [142]. The dppn complex binds more strongly than the dppz variant, but both are weaker binders than [Ru(bipy)_2_(dppz)]^2+^. Corresponding luminescence spectroscopy titrations revealed a very strong emission enhancement for [Ir(ppy)_2_(dppz)]^+^ upon DNA addition, attributed to hydrophobic shielding of the dppz ligand from water‐mediated quenching [143]. [Ir(ppy)_2_(dppn)]^+^ likely possesses significant dppn‐centred ^3^IL character to its emission (rather than ^3^MLCT) [144] but showed more subtle modulation of emission upon DNA titration. More recent studies by others have shown that the dppn ligand can be susceptible to photooxidation in certain Ir(III) complexes, which must be a consideration for future biological applications [145]. Furthermore, the dppz‐based, biotin–functionalised complex **9** (Figure 13) exhibited selective binding with avidin, but no strong interactions with DNA, presumably sue to the steric bulk of the biotin moiety that prevents intercalation.

In further work, Lo showed that dendritic arrays integrating multiple cyclometalated Ir(III) luminophores did not interact strongly with ct‐DNA, but condensed plasmid DNA via electrostatic attractions, which was confirmed through agarose gel retardation assays [146].

**FIGURE 14:**
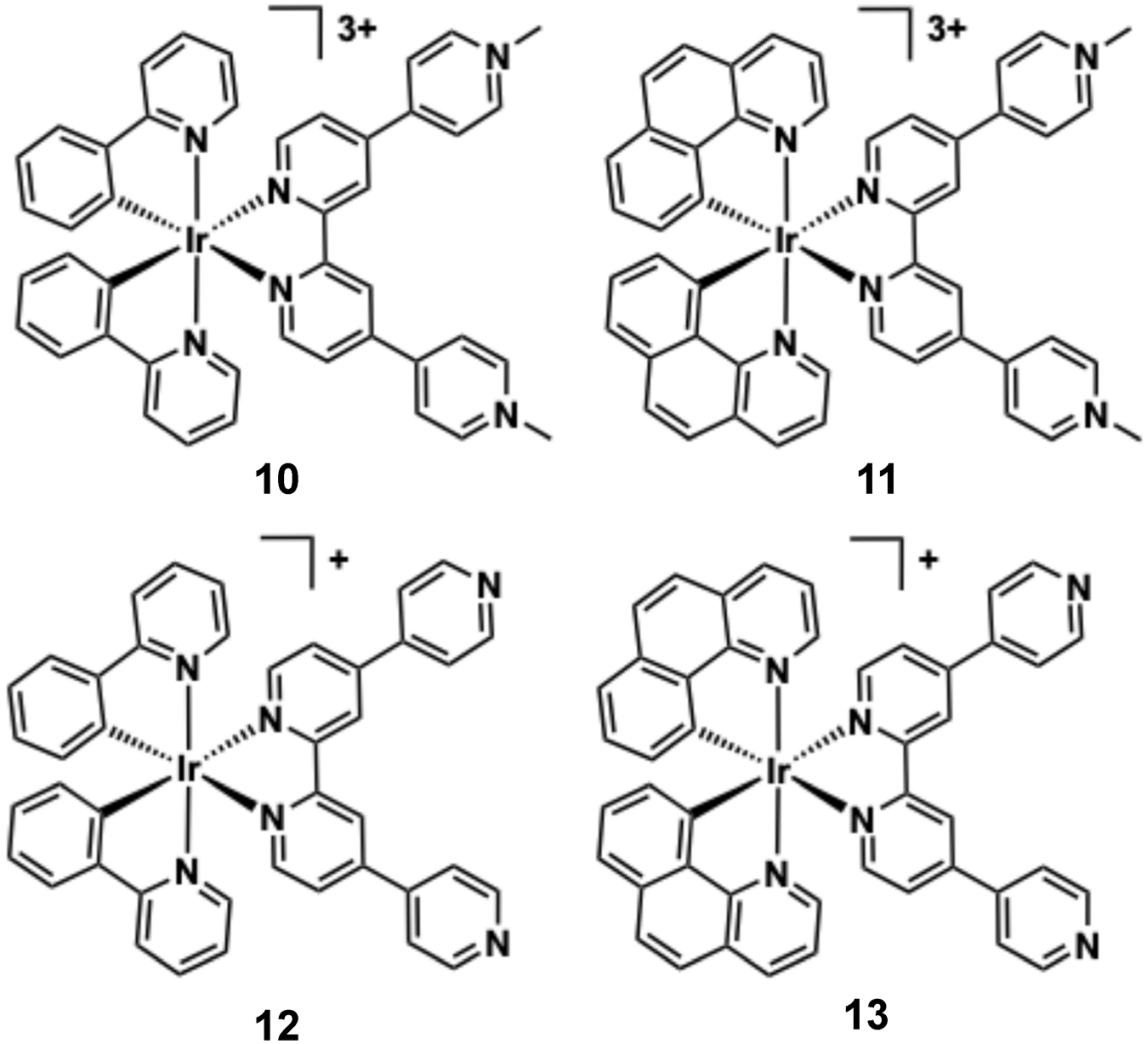
Structures of complexes **10–13**.

Thomas and co‐workers have studied the behaviour of related cyclometalated Ir(III) [Ir(C ^ N)_2_(N ^ N)]^n+^ systems **10**‐**13** which bear quaterpyridyl (qtpy) or cationic methylated quaterpyridyl (Me_2_qtpy^2+^) co‐ligands (Figure 14) that are close analogues of complexes **4** and **5,** discussed earlier. For context, monocationic **12** and **13** (i.e. non‐methylated qtpy) displayed poor water solubility, which precluded meaningful DNA measurements [147]. The tricationic complexes **10** and **11** (with Me_2_qtpy^2+^), however, had suitable solubility and exhibited strong minor groove DNA binding (*ca.* 3.5 × 10^6^ M^−1^ for **10** and **11**). The groove binding was characterised by hyperchromic absorption changes and negative viscosity shifts, in contrast to the intercalative behaviour seen in their Ru(II)/Re(I) analogues [83, 84]. UV–vis titrations and viscosity assays established their binding affinities and modes.

**FIGURE 15:**
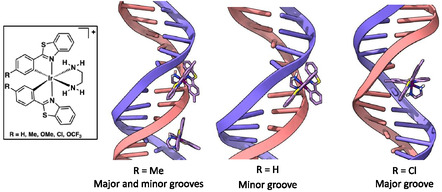
Structures of complexes **14**–**18** (left, inset) and top calculated DNA binding modes using Autodock Vina [150] using open d(ATCGAGACGTCTCGAT)_2_ structure.

These studies emphasised how critical water solubility is for cyclometalated Ir(III) complexes and their study with DNA. Aromatic ligands are, by their nature, hydrophobic and without an increase in overall charge many cyclometalated complexes simply do not possess sufficient solubility. An alternative approach is to consider the use of hydrophilic ancillary ligands which can promote H‐bonding interactions [148], overcoming the limitation of a mono‐cation. In an approach inspired by Lo and co‐workers who had previously reported diamine co‐ligands in organometallic Ir(III) species [149], our group developed a series of mono‐substituted 2‐phenylbenzothiazole cyclometalated Ir(III) complexes which combine an ethylenediamine (en) co‐ligand, [Ir(C ^ N)_2_(en)]^+^ **14**‐**18** (R = H, Me, OMe, Cl, OCF_3_, respectively) and a chloride counter ion to promote water solubility (Figure 15) [81]. These green‐emissive complexes (*λ*_em_ = 529–540 nm, Φ_em_ up to 12%) exhibit luminescence enhancements up to 98% upon DNA binding, presumably due to shielding of the complex from quenching phenomena (the key excited state is localised on the 2‐phenylbenzothiazole ligands). Supporting time‐resolved emission measurements confirmed this through notable extensions in luminescence lifetime in the presence of DNA; for example, addition of DNA to **15** (R = Me) enhanced the lifetime from 0.399 to 0.807 µs) indicative of triplet excited state shielding. Biophysical studies (UV–vis titrations and ITC) revealed dual binding modes: i) a high‐affinity groove binding (*K*_*b*_ = 10^7^–10^8^ M^−1^), with groove preference (minor/major) influenced by substituent bulk (as noted in other systems [151, 152, 153]), and ii) weaker electrostatic interactions (*K*_*app*_ ≈ 10^5^ M^−1^). Docking simulations (Autodock Vina) suggest a non‐intercalative binding mode where **14** and **15** bind the minor groove, **17** the major groove. Hydrophobic substituents (e.g. ‐OCF_3_ in **18**) enhanced DNA affinity but reduced solubility. During the study, complex aggregation (often overlooked in the analysis of DNA binding complexes) was noted (critical aggregation concentration of 2.1 µM for **14**) in high‐salt buffer (50 mM NaCl) but suppressed by using 5 mM NaCl. ITC data confirmed entropy‐driven groove binding (e.g. Δ*H* ≈ −3.3 kJ mol^−1^, −TΔ*S* = − 44.5 kJ mol^−1^ for **14**), consistent with dehydration effects [154]. In summary, these relatively simple organometallic complexes interact strongly with DNA despite lacking extended planarity ligands.

**FIGURE 16:**
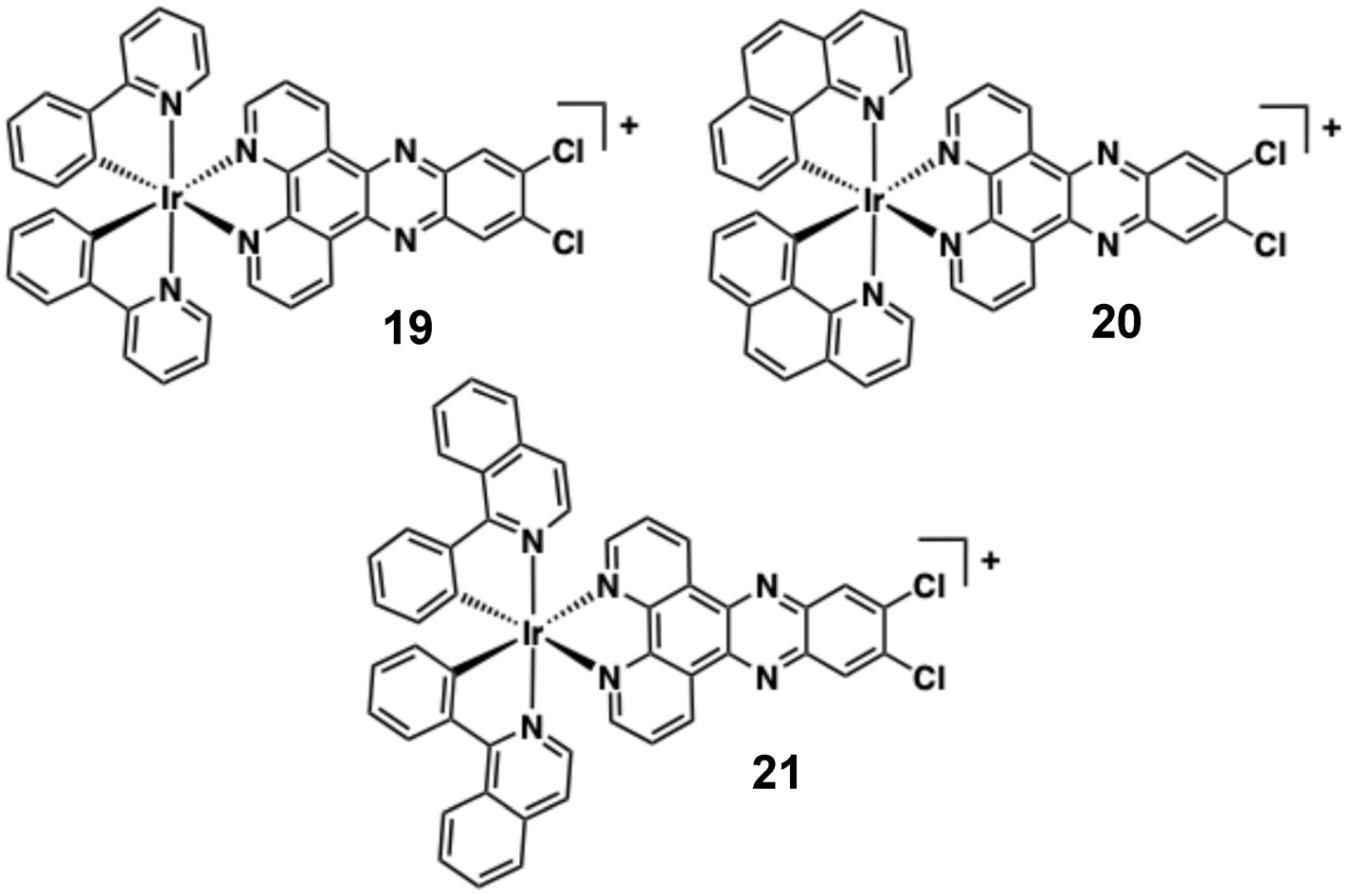
Structures of complexes **19**–**21**.

In an iteration of dppz‐based ligand systems, Wang and co‐workers investigated the DNA‐binding properties of complexes **19**, **20**, and **21** (Figure 16) [57], which are monocationic [Ir(C ^ N)_2_(N ^ N)]^+^ species. The complexes integrate a dichloro‐substituted dppz ligand (the planar nature of the ligand facilitates strong intercalative binding via minor‐groove insertion) and vary according to the precise conjugated detail of the C ^ N ligand. Molecular docking and dynamics simulations suggested significant DNA structural distortions, with base‐pair separation increasing from 3.39 to 6.87 Å, accompanied by helix distortion. Despite the apparent hydrophobic nature of these species, spectroscopic characterisation revealed DNA binding affinities of 3.19–4.88 × 10^5^ M^−1^. UV–vis titrations revealed characteristic hypochromism (up to 27%) and bathochromic shifts, while fluorescence quenching and viscosity measurements confirmed the intercalative binding mode. The observed DNA binding affinity trend (**19** > **20** > **21**) correlated with both the steric profile of the C ^ N ligands (ppy < bzq < piq) and the complexes’ cytotoxicity.

Recent studies on cationic, heteroleptic phenyl‐1*H*‐pyrazole Ir(III) complexes, [Ir(C ^ N)_2_(N ^ N)]^+^ (where N ^ N = 2‐di(pyridyl)ketone, 2,2′‐biimidazole or 2‐(2′‐pyridyl)benzimidazole) has also shown that the nature of the N ^ N co‐ligand can influence the strength of an intercalative interaction with ct‐DNA. However, the complexes were generally non‐ or weakly emissive under ambient conditions which limit bioimaging applications [155].

**FIGURE 17:**
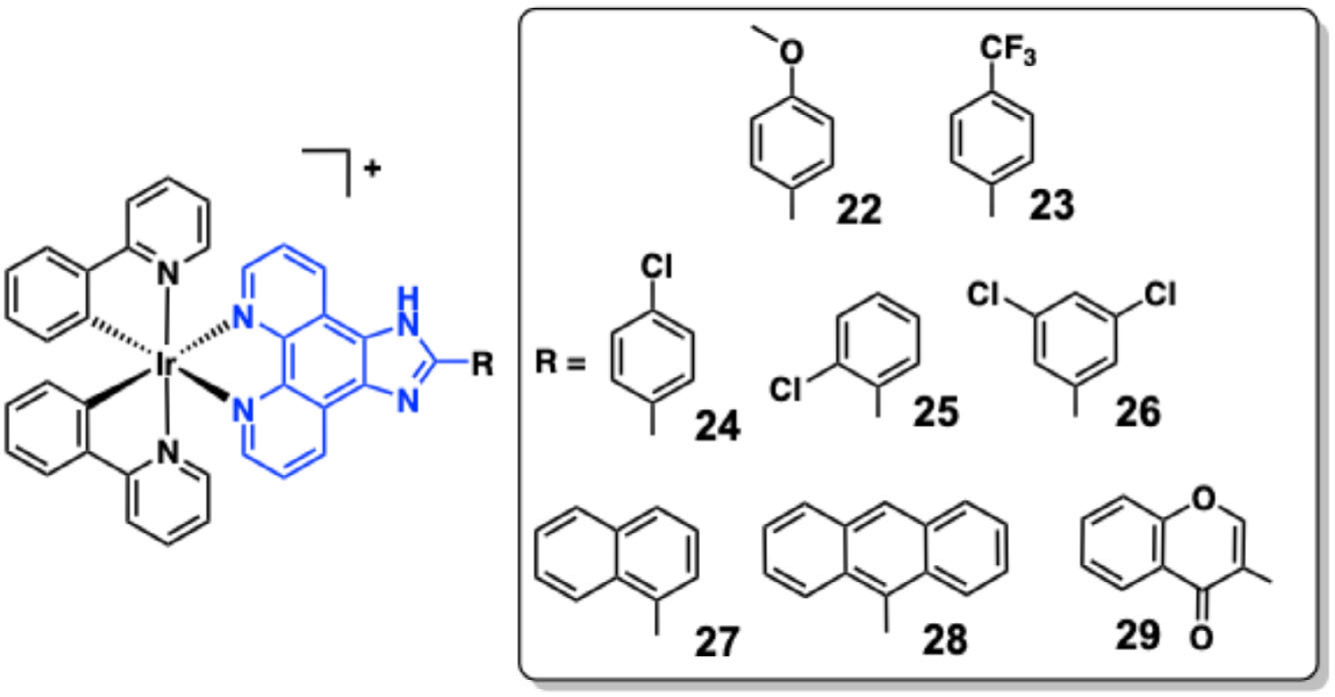
Structures of complexes **22–29**. The imidazo[4,5‐*f*]−1,10‐phenanthroline unit is highlighted in blue; R groups shown inset.

The conjugated structure of imidazo[4,5‐*f*]−1,10‐phenanthroline is a convenient ligand framework for the development of functional luminescent molecules and biologically active species [156] and is attractive due to its synthetic accessibility (usually yielded in a single step from 1,10‐phenanthroline‐5,6‐dione) [157, 158, 159]. This ligand system has been exploited in the development of related cyclometalated Ir(III) complexes (**22**‐**26,** Figure 17) and shown to promote binding to G‐quadruplex DNA sequences, leading to an enhancement in ^3^MLCT/^3^LLCT emission [160]. Interestingly these complexes were integrated into an electrochemiluminescent‐based G‐quadruplex assay.

A number of research groups have developed chromophore‐functionalised systems to promote DNA binding and provide additional signal output to inform upon the nature of binding. Paira and co‐workers prepared three cyclometalated Ir(III) complexes, **27**–**29**, also based upon an elaborated imidazo[4,5‐*f*]−1,10‐phenanthroline structure that incorporate naphthalene, anthracene, and chromone moieties, respectively, as ROS‐generating photodynamic agents (Figure 17) [80]. UV–vis titrations with ct‐DNA revealed significant hypochromism for all three complexes, with **27** and **29** also showing moderate bathochromic shifts consistent with intercalative binding, while **28** exhibited strong DNA affinity without a notable red‐shift. This behaviour was attributed to the planar anthracene ligand in **28**, which facilitates efficient insertion between DNA base pairs [64]. Ethidium bromide (EB) displacement assays confirmed intercalation, with **28** displaying the highest apparent binding constant (*K*_*app*_ = 3.2 × 10^6^ M^−1^). The intrinsic binding constants (*K*_*b*_) ranged from 0.73 to 1.59 × 10^5^ M^−1^, reflecting the impact of the ancillary ligand structure on DNA interactions. Molecular docking supported an intercalative binding mode, with **28** exhibiting a binding energy of −10.4 kcal/mol, stabilised by π‐π stacking and hydrophobic contacts with DNA bases [161]. With dual ROS generation and strong DNA binding, **28** is suggested as a promising phototherapeutic candidate. Closely related Ir(III) complexes with terminal phenyl‐NH_2_ groups on the imidazo[4,5‐*f*]−1,10‐phenanthroline ligand have also demonstrated DNA binding and anticancer cytotoxic behaviour in vivo [162].

**FIGURE 18:**
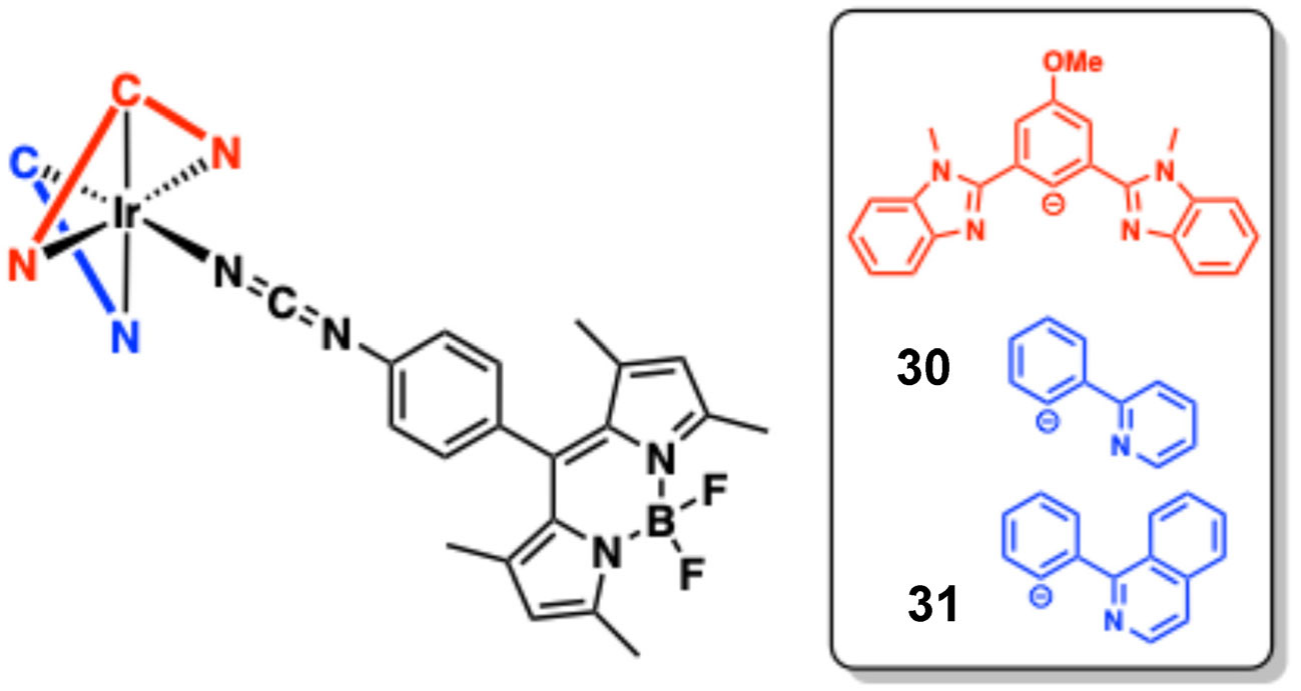
Structures of BODIPY appended complexes.

Two cyclometalated Ir(III) complexes (**30** and **31**) adorned with a *meso*‐phenylcyanamide BODIPY co‐ligand (**L** ^**BODIPY**^) have been studied with respect to phototherapeutic effects. The proposed formulation of the complexes studied, [Ir(N ^ C ^ N)(N ^ C)(**L** ^**BODIPY**^)] allowed some structural variety (via C ^ N ligands) to be assessed (Figure 18). Despite their charge neutrality, the complexes were evaluated in Tris buffer and shown to be effective binders for ct‐DNA binding. UV–vis titrations revealed *K* of 4.39 ± 0.2 × 10^5^ M^−1^ and 1.31 × 10^6^ M^−1^, respectively, and supporting EB displacement assays suggest partial intercalation of the complexes involving the BODIPY chromophoric portion [163]. Upon photoirradiation at 500 nm, the complexes also displayed cleavage of SC DNA to its nicked circular (NC) form (100 μM complex for 1 h).

**FIGURE 19:**
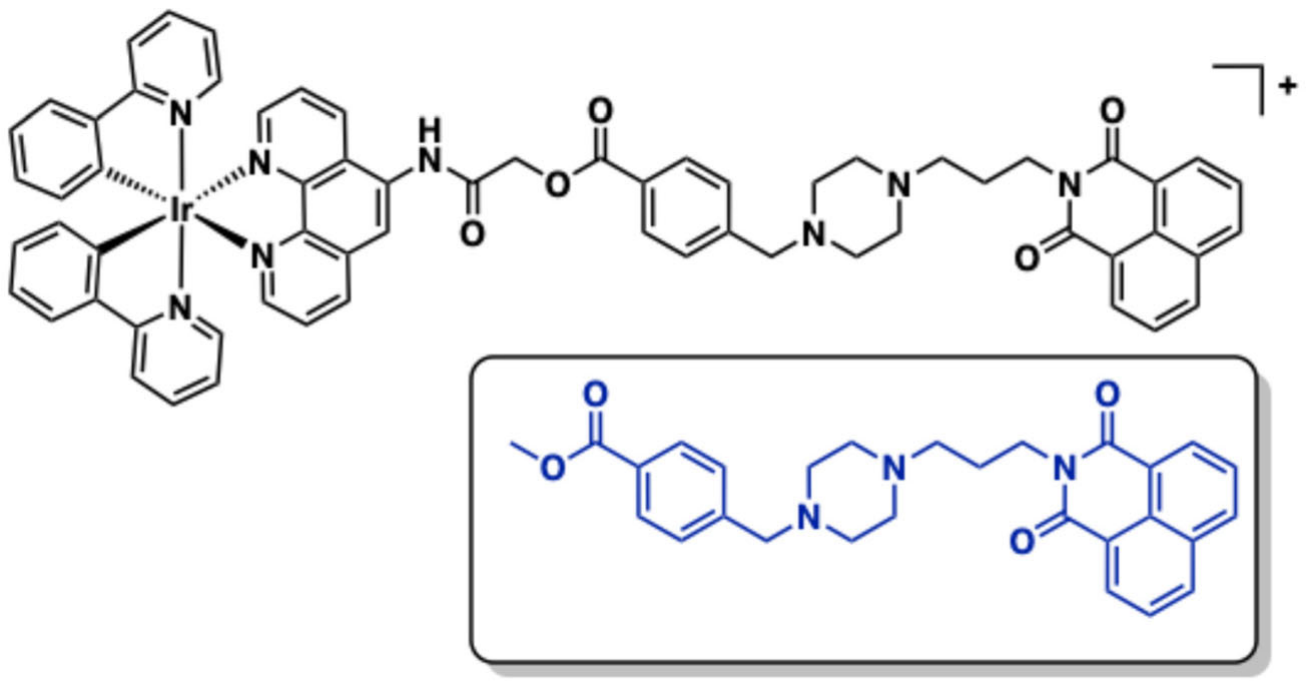
Structure of complex **32** (reference 1,8‐naphthalimide shown inset).

Ma and co‐workers linked an emissive cyclometalated Ir(III) complex with a 1,8‐naphthalimide groove‐binding motif, yielding a hybrid complex **32** (Figure 19) to target G‐quadruplex (G4) DNA recognition [82]. This design retained the photophysical properties of [Ir(ppy)_2_(phen)]^+^ and showed stronger G4 affinity and selectivity than a model 1,8‐naphthalimide compound (inset, Figure 19) [164]. FRET melting assays [165] demonstrated a 13°C stabilisation of G4 DNA by **32** and luminescence titrations revealed a 3.5‐fold luminescence enhancement for G4 over ss‐DNA, driven by groove‐binding‐induced environmental changes around the Ir(III) complex. Time‐resolved emission spectroscopy was used to discriminate the long‐lived phosphorescence of **32** and CD confirmed G4 formation upon AGR2‐induced structural switching [166]. Molecular docking studies stressed the hydrophobic interactions between the pendant 1,8‐naphthalimide unit and G4 loop/groove regions. Therefore, by integrating a groove‐binding motif, **32** achieved enhanced G4 selectivity and affinity, enabling prospective biosensing applications.

**FIGURE 20:**
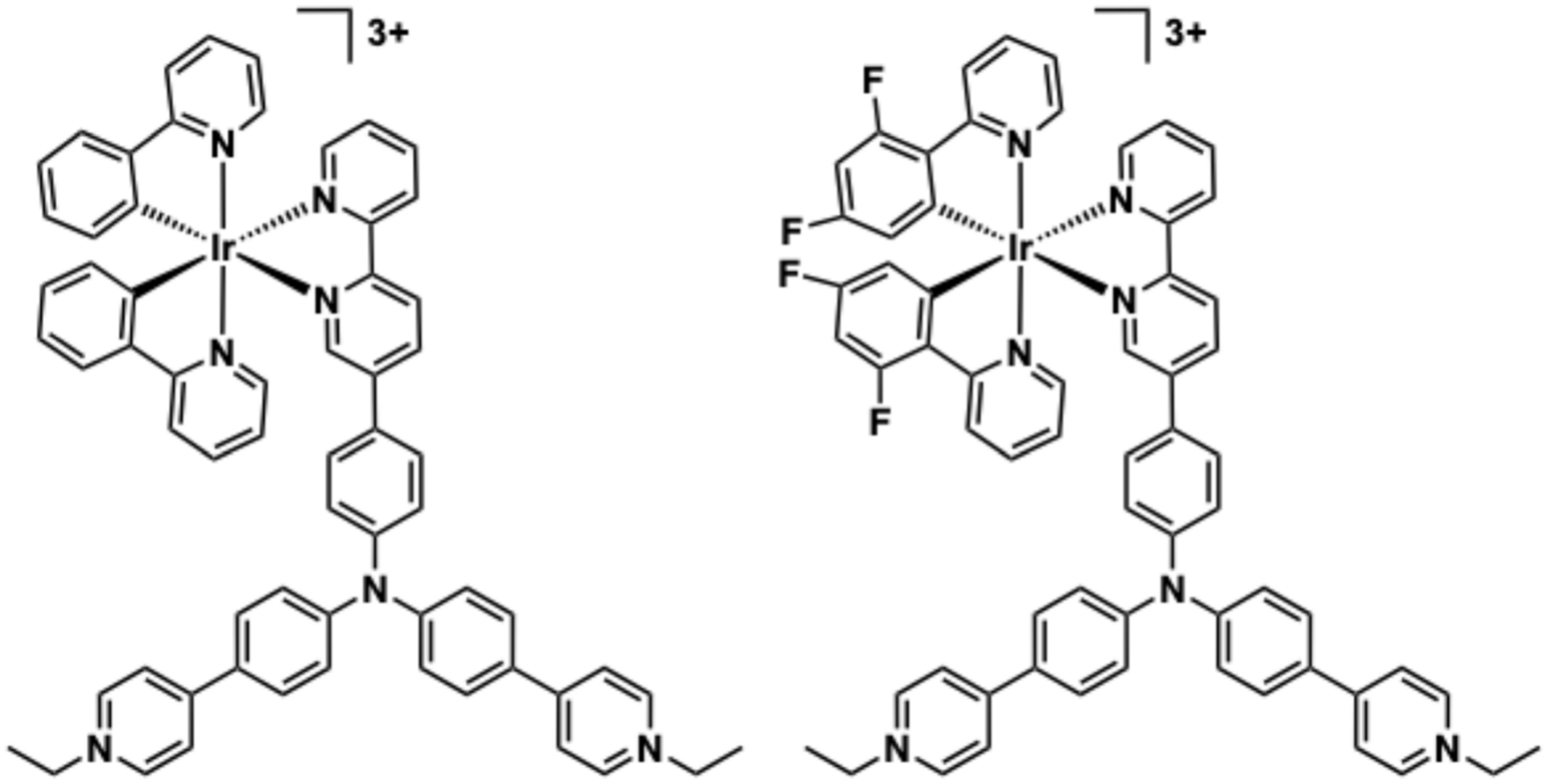
Structures of complexes **33** and **34**.

Cyclometalated Ir(III) complexes **33** and **34**, based upon a [Ir(ppy)_2_(bipy)]^+^ core with dicationic triphenylamine substituents at the 5‐position of the bipy ligand (Figure 20), have been studied by Mao and co‐workers. Both complexes bind to double‐stranded DNA (*K*_*b*_ = 9.73 ± 0.75 × 10^5^ M^−1^ for **33**; 1.63 ± 0.61 × 10^6^ M^−1^ for **34**) and the human telomeric G‐quadruplex (Tel26), as determined by UV–vis titrations [167]. Luminescence studies also revealed emission enhancement upon DNA binding implying some extent of shielding (primarily from ^3^MLCT/^3^LLCT states) from quenching phenomena. While fluorination of the coordinated phenyl ring in **34** slightly increased its phototoxicity (IC_50_ = 12 nM vs. 17 nM for **33**), both complexes exhibited similar DNA interaction modes: minor groove binding to ds‐DNA (hydrophobic/electrostatic) and G‐tetrad stacking in Tel26 (π‐π interactions). **33** and **34** induced DNA double‐strand breaks (γH2AX), cytosolic DNA leakage [168], and subsequent AIM2 inflammasome activation [169], culminating in GSDMD‐mediated pyroptosis [170]. Overall, both complexes induce immunogenic cell death and the use of fluorine substituents boosts efficacy without altering binding characteristics.

**FIGURE 21:**
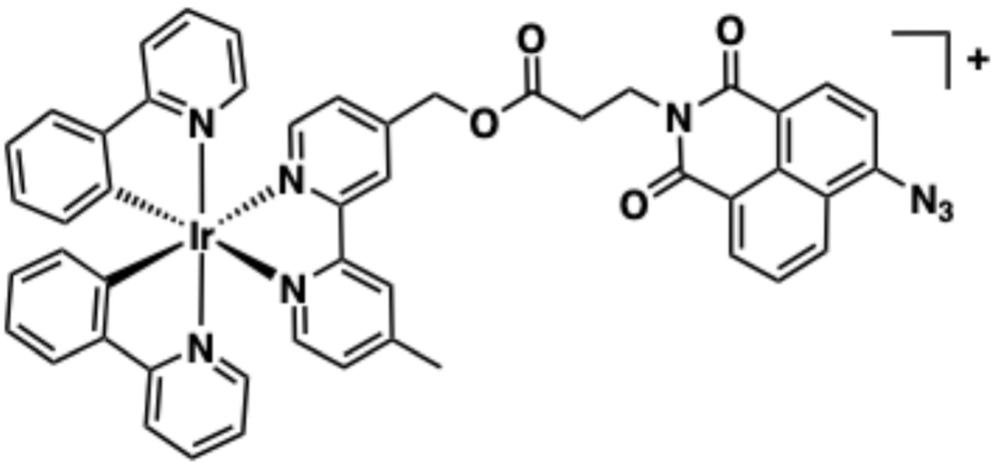
Structure of complex **35**.

The same research group designed a H_2_S‐responsive luminescent probe **35** containing an azide‐functionalised 1,8‐naphthalimide ligand that acts as both a mitochondrial DNA (mt‐DNA) intercalator and a cancer‐specific therapeutic agent (Figure 21) [171]. Molecular docking studies suggest **35** strongly intercalates into mt‐DNA through π‐π stacking interactions with nucleobases, stabilised via hydrogen bonds with the phosphate backbone. Modest DNA binding affinity (*K*_*b*_ = 9.89 × 10^3^ M^−1^) was calculated using the double reciprocal linear equation (*R* = 0.998), which increased upon H_2_S activation [172]. UV–vis titrations showed hypochromicity and a red shift upon DNA addition, supporting an intercalative binding mode, while luminescence spectroscopy (via competitive EB displacement assays) demonstrated 75% quenching. Cellular studies using PicoGreen staining visualised **35** undergoing selective mt‐DNA binding [173], further verifying its strong interaction with DNA.

**FIGURE 22:**
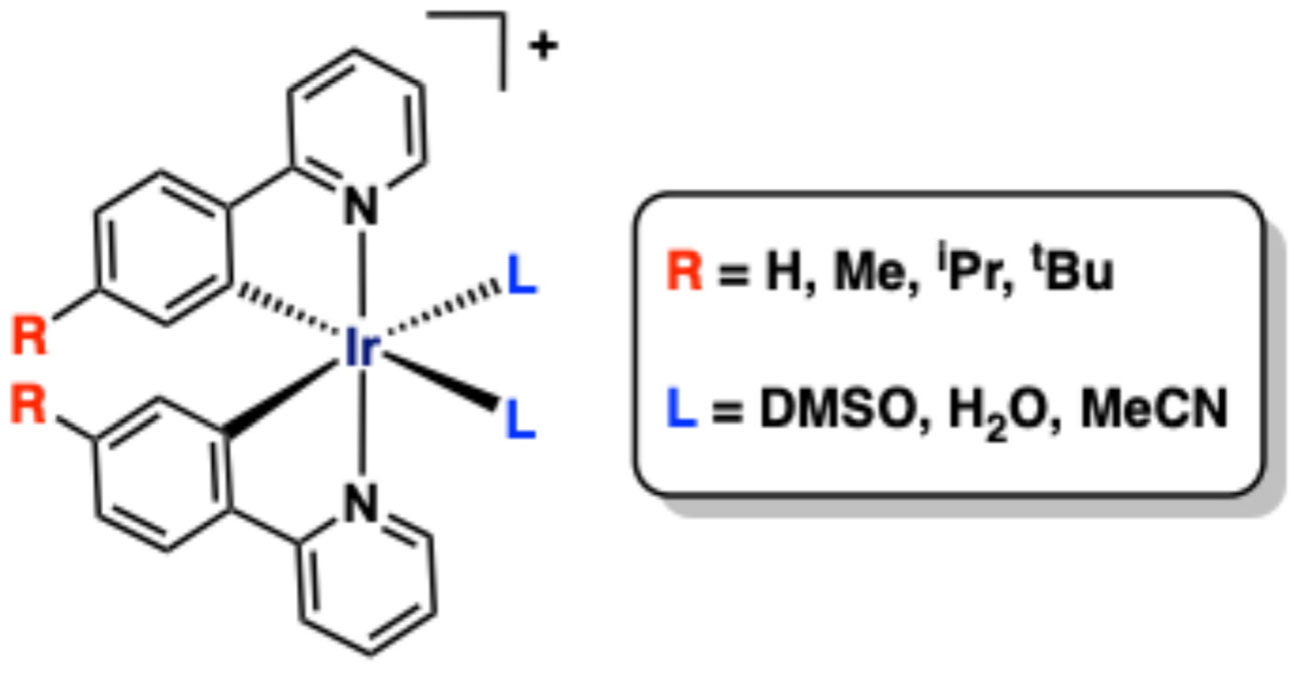
Core structure **36** and derivatives thereof.

The discussion so far has focused upon non‐covalent binding interactions with DNA. However, researchers have investigated the possibility of covalent interactions using *bis*‐solvated cyclometalated Ir(III) complexes of the basic formula [Ir(C ^ N)_2_(L)_2_]^+^. Structure **36** (Figure 22) shows the core features of these different cationic species with two cyclometalated ppy ligands (where R tunes lipophilicity) and two monodentate solvent ligands completing the coordination sphere [174]. The complexes are weakly or non‐emissive, but in a series of biological studies, encompassing biomolecule interactions, cytotoxicities, and cell imaging work, the complexes show some interesting attributes. In general, the complexes reveal a strong luminescent enhancement upon addition of histidine or histidine rich proteins. Presumably, this is due, despite the kinetic inertness, to ligand solvent substitution at the Ir(III) centre. The authors were able to utilise this in a cell imaging context showing that advantageous “switch on” luminescence was demonstrated following a rapid energy dependent membrane pathway and selective nuclear localisation [175]. However, no profound modulation of luminescence was noted upon addition of ct‐DNA, implying that similar ligand substitution reactions do not occur and neither non‐covalent, nor covalent DNA interactions are operative for these solvated complexes.

## 8 DNA Binding Behaviour of Di‐ and Multi‐Metallic Arrays Incorporating Ir(III)

**FIGURE 23:**
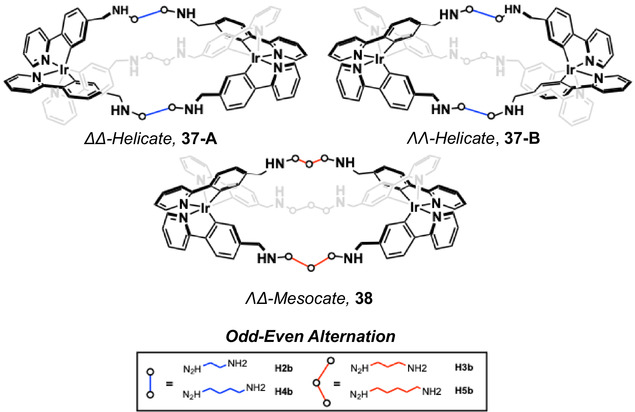
Structures of complexes **37**(**A/B**) and **38**.

The consideration of molecular arrays encompassing more than one metal ion can allow researchers to address a variety of challenges related to DNA binding. Duan and co‐workers developed dimetallic Ir(III) assemblies **37** and **38** for targeting mitochondrial DNA in cancer cells (Figure 23) [176]. Interestingly, the complexes were based upon interlinked ‘*fac*‐[Ir(ppy)_3_]’ units assembled via imine coupling of aldehyde‐functionalised ppy ligands and diaminoalkane spacers; imine reduction then yielded more hydrolytically compatible amine‐linked structures. Despite a formally charge neutral state, the amine sites are available for H‐bonding and protonation and promote water solubility. The odd‐even character and length of the diamine linkers dictated the stereochemistry, affording either helicate **37** (**H2b** and **H4b**, **A**/**B**) or mesocate **38** (**M3b** and **M5b**) [177, 178, 179, 180]. The mesocates exhibited stronger DNA‐binding affinity and superior PDT performance compared to the helicates. DNA interaction was investigated through EB displacement assays (quantifying binding affinity), molecular docking (simulating binding modes and energies), and DNA photocleavage experiments (assessing photoinduced damage efficiencies). Among the series, mesocate **38** (**M5b**) displayed the highest binding affinity for DNA (*K*_*app*_ = 1.12 × 10^7^ M^−1^) with preferential minor‐groove binding, leading to enhanced apoptosis induction upon light irradiation. This work demonstrates that controlling Ir(III) metallohelix stereochemistry can enhance DNA selectivity and PDT effectiveness [181, 182].

**FIGURE 24:**
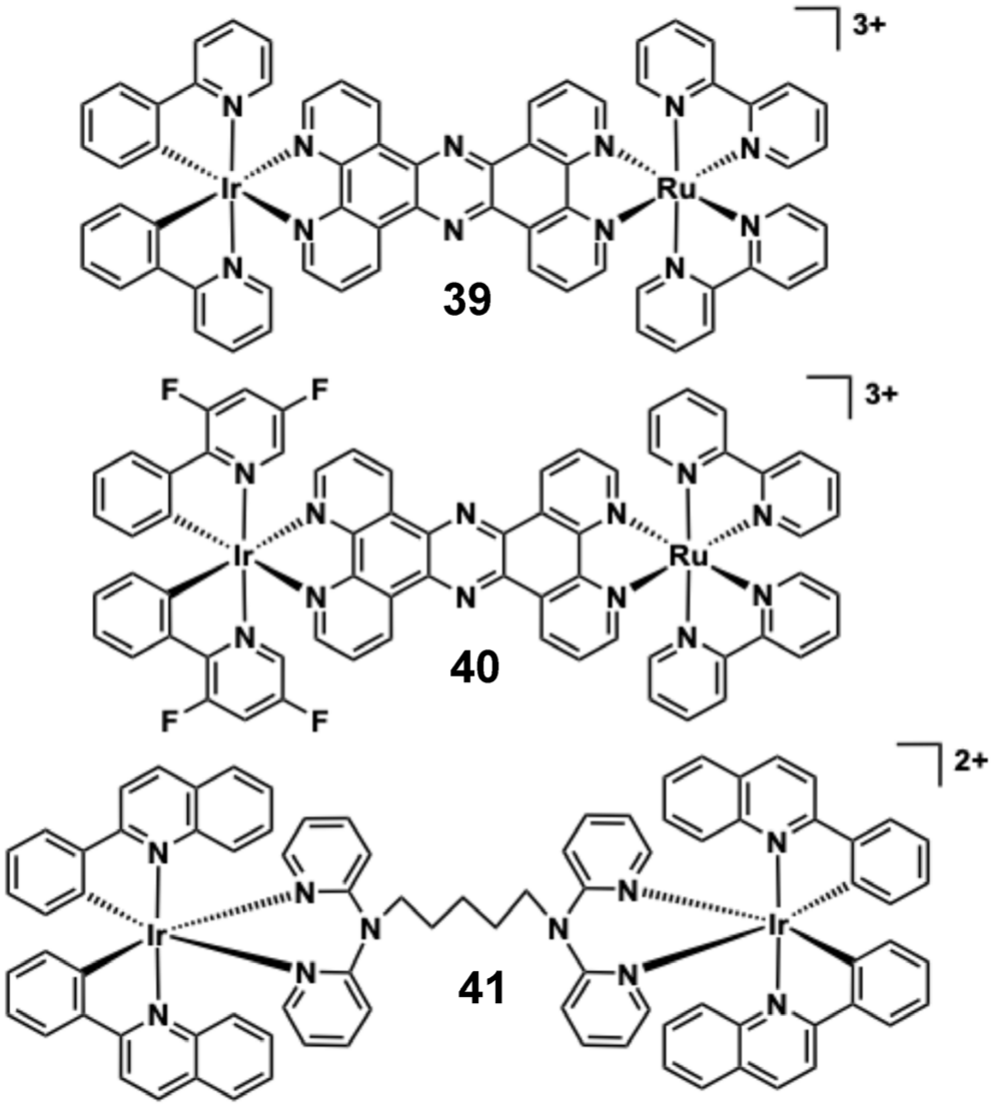
Structures of bimetallic complexes **39**–**41**.

Thomas and co‐workers reported heterobimetallic, tricationic Ir(III)‐Ru(II) complexes **39** and **40** (Figure 24) that are based upon a bridging tetrapyrido[3,2‐*a*:2′,3′‐*c*:3″,2″‐*h*:2′′′,3′′′‐*j*]phenazine (tpphz) ligand; the complexes were isolated as their chloride salts to maximise water solubility [183]. Both complexes encompass ‘[Ir(ppy)_2_(N ^ N)]’ units and differ according to the cyclometalating ligand at Ir(III); **40** possesses a difluorinated ppy variant. Both complexes are luminescent in the red region and closely comparable to the properties of the Ru(II)‐Ru(II) analogue, implying that emission originates from the Ru(II) ^3^MLCT portion of the dimer. The quantum yields were noted as rather poor, especially in water. These heterometallic species exhibit different DNA‐binding behaviour compared to the homonuclear Ru(II)‐Ru(II) analogue. Whereas the Ru(II)‐Ru(II) systems can bind through intercalation and non‐intercalative modes (the latter, interestingly, still giving a light switch effect [184]), the Ir(III)‐Ru(II) complexes favourably engage in groove‐binding, as demonstrated by DNA‐induced luminescence enhancements and bathochromic shifts. The McGhee‐von Hippel model gave DNA binding constants of *K* = 1.49 × 10^6^ M^−1^ for **39** and 3.38 × 10^5^ M^−1^ for **40**, with the higher affinity of **39** attributed to its more favourable charge distribution for DNA interaction. Importantly, incorporation of Ir(III) into the structures helped address a key limitation of Ru(II)‐Ru(II) systems, which displayed inefficient nuclear localisation. Cell imaging work (HeLa cell line) using confocal fluorescence microscopy (using an excitation wavelength of 488 nm, which helps minimise autofluorescence) showed the fluorinated complex **40** combined enhanced membrane permeability (likely promoted by the lipophilic fluorinated ligand) with precise nuclear targeting. Overall, this study demonstrated that heterometallic Ir(III)‐Ru(II) architectures provide greater tunability in DNA recognition and, via favourable photophysical attributes, can yield biologically compatibility systems with distinct advantages over homometallic Ru(II) analogues.

Ma and co‐workers have shown that the linker unit in homobimetallic Ir(III) species does not need to be rigid to promote DNA binding. For example, bimetallic **41** (linked via *N* ^1^,*N* ^1^,*N* ^5^,*N* ^5^‐tetra(pyridin‐2‐yl)pentane‐1,5‐diamine) was shown (as validated by a FRET melting assay) to be a selective binder for G‐quadruplex DNA over ds‐DNA and ss‐DNA. The enhanced luminescence characteristics are governed by the cyclometalating 2‐phenylquinoline ligands, which may also impart additional hydrophobicity which is advantageous as a G‐quadruplex probe (the analogous 2‐phenylpyridine based dimer did not perform as well). The flexible alkyl chain may also allow a conformational optimum for G‐quadruplex DNA binding; this allowed the development of a HIF‐1α label free assay [185].

## 9 DNA Binding Behaviour of Half‐Sandwich Metallocene Ir(III) Complexes

**FIGURE 25:**
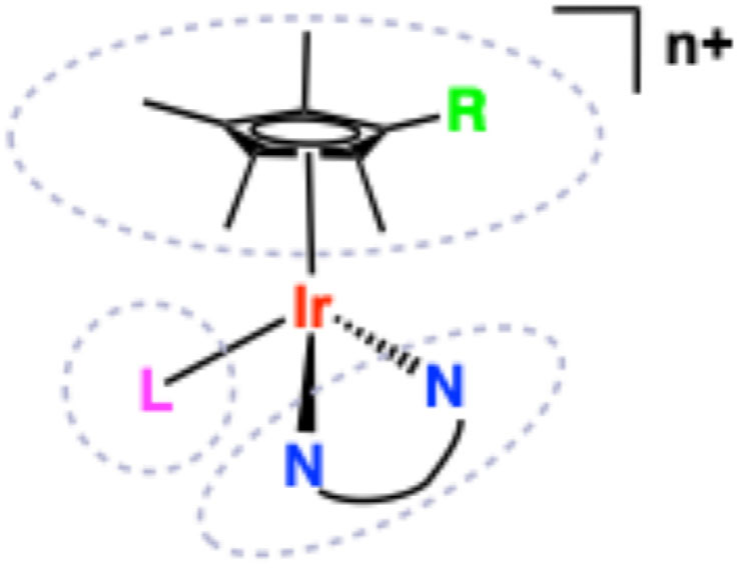
General arrangement of organo‐Ir(III) piano stool complexes. Three key ligand features: i) axial, R‐substituted cyclopentadienyl; ii) monodentate, L; ii) bidentate N ^ N.

Organoiridium species can adopt a number of oxidation states (+1, +3, +4) and generally fall into categories of complex with coordination numbers of 4 or 6. Having discussed the DNA interactions of (generally photoluminescent) octahedral Ir(III) complexes, it is important to recognise that other classes of Ir(III) species have been studied for their biological behaviour and it is therefore pertinent to highlight those studies which have included DNA binding as part of their investigations. For example, organo‐Ir(III) species that adopt a half‐sandwich structure are often referred to as piano stool complexes: an electron rich cyclopentadienyl ligand (for example, pentamethylcyclopentadienyl, Cp*) occupies an axial position (and helps stabilise Ir^3+^) and three remaining coordination sites can be addressed by co‐ligands (which can tune biological activity). These pseudo‐octahedral complexes [(*η* ^5^‐Cp*)Ir(N ^ N)(L)]^0/n+^ (Figure 25) can be synthesised in a stepwise manner and, together with other metallocenes, have been extensively studied for their anticancer properties.

Within this general formulation (where N ^ N = bidentate ligand with nitrogen donors but can also be oxygen and/or carbon donor atoms; L = monodentate Cl or pyridine) adaptation of the Cp* ligand can include extended hydrophobic arenes or applying chelating ligands can help promote intercalation into DNA [186, 187]. It is also important to note that the coordinated chloride site in [(*η* ^5^‐Cp*)Ir(N ^ N)Cl]^n+^ can be hydrolysed in aqueous solution (and typically remains “Ir‐OH_2_” rather than “Ir‐OH” at physiological pH) which becomes important when considering the overall charge of the complex and covalent interactions with nucleobases. While this class of complex have been studied with respect to excellent biological and potential therapeutic activity, they do not possess the same advantageous photophysical properties as described earlier for either polypyridine or cyclometalated Ir(III) species, which limits their diagnostic capacity especially in the context of live cell confocal fluorescence microscopy.

Nearly 20 years ago, a number of publications by Sheldrick and co‐workers showed that organo‐Ir(III) complexes could be developed with classical DNA binding ligands such as dppz. Structure **42** has the general formulation [(*η* ^5^‐Cp*)Ir(N ^ N)(L)]^n+^ (where N ^ N = dpq, dppz, dppn; L = Cl, (NH_2_)_2_CS, (NMe_2_)_2_CS), and these varied complexes were studied for their DNA interactions using UV–vis titrations, viscometry, and CD spectroscopy [188]. The work showed that intercalation with DNA is possible via the planar N ^ N ligand, with enhancements observed for the rate of chloride hydrolysis once bound to DNA. Interestingly, for the dppn species, the precise mode of binding to DNA was dictated by the monodentate co‐ligand.

**FIGURE 26:**
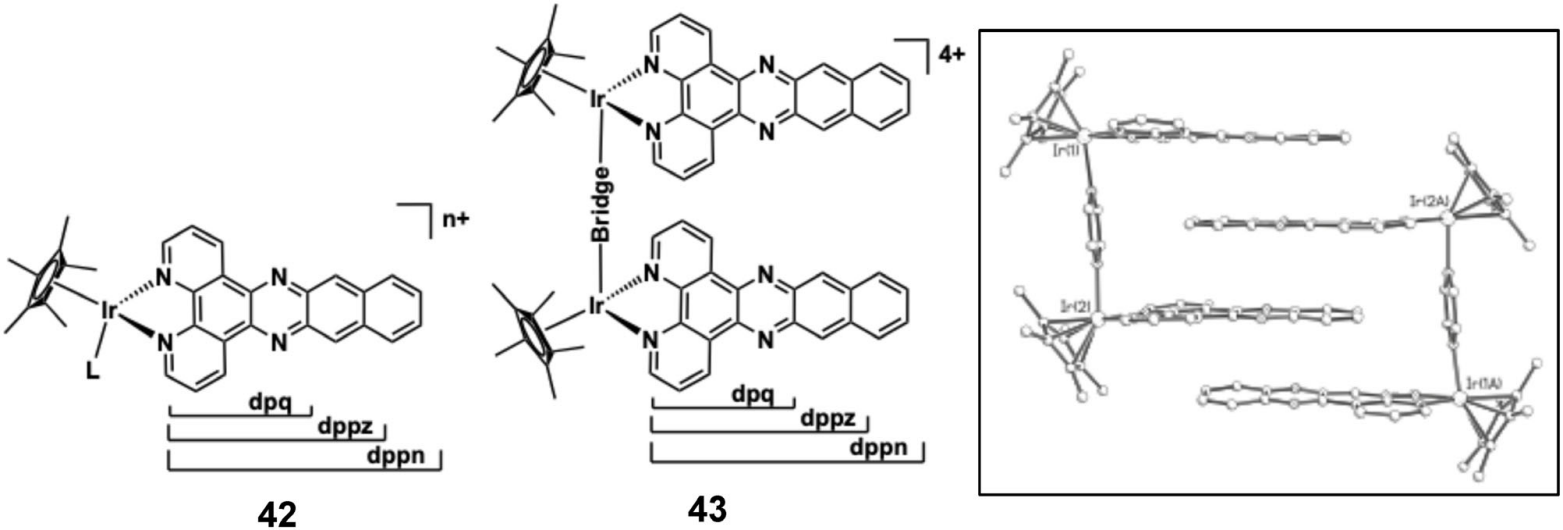
Core molecular structures of **42** and bimetallic variants **43**. X‐ray crystal structure of the pyrazinebridged, dppz‐based Ir(III) dimer shown inset. Reproduced and with permission. Copyright 2009, Elsevier.

Closely related work has shown how bridging ligands (pyrazine, 4,4’‐bipyridine) can link two organo‐Ir(III) complexes together to give structures based on **43** (Figure 26) [189]. The aim was to increase overall cationic charge, promote complementary DNA binding behaviour, and enhance biological activity. UV–vis and CD spectroscopies, thermal denaturing experiments, viscosity assessments, and NOESY NMR studies confirmed that the dppz variants worked best: [{(*η* ^5^‐Cp*)Ir(dppz)}_2_(μ−4,4′‐bipy)]^4+^ strongly interacts with DNA. DNA‐binding studies showed that this species is a potent intra‐strand *bis*‐intercalator, inserting both dppz units between the C3G18/G4C17 and T5A16/A6T15 base pairs, as evidenced by a large thermal stabilisation (ΔT_m_ = +19°C), a viscosity slope of 1.4, and characteristic NMR NOE patterns. In comparison, the shorter pyrazine‐bridged analogue reveals mono‐intercalation (ΔT_m_ = +12°C, viscosity slope = 0.7). Optimising the bridging ligand and a suitably sized intercalator are critical for effective *bis*‐intercalation [1].

**FIGURE 27:**
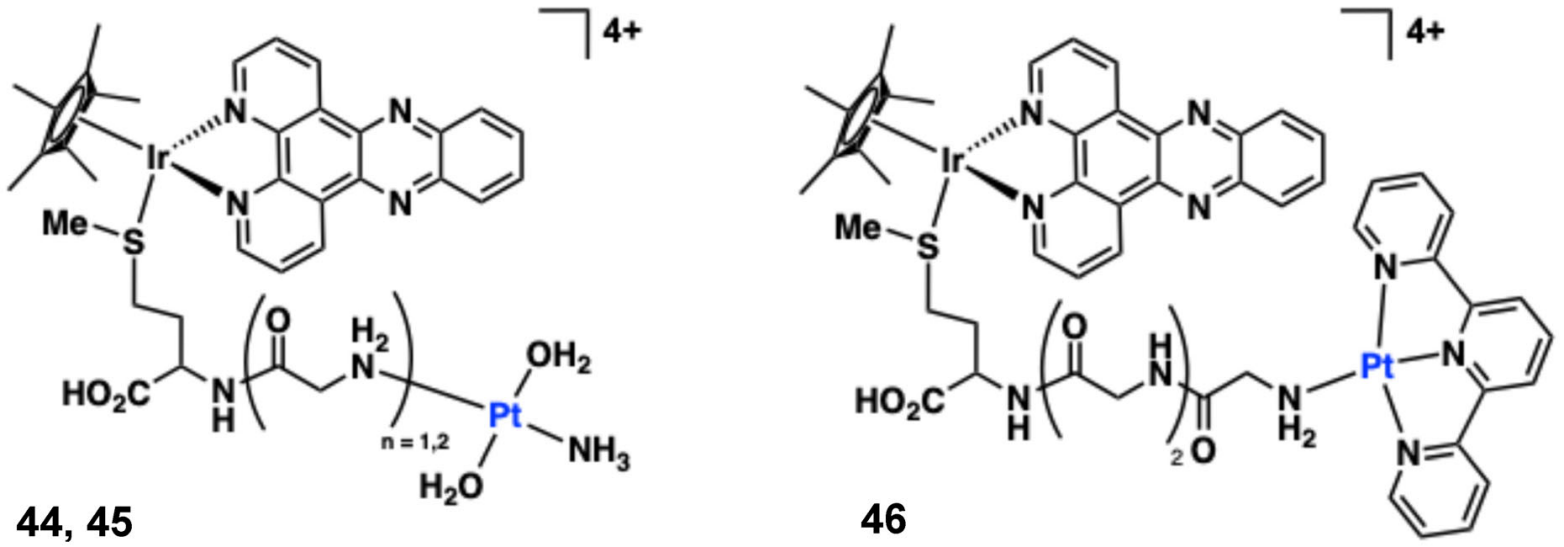
Structures of heterometallic Ir(III)/Pt(II) complexes **44–46**.

In an evolution of these structures, heterobimetallic Ir(III)‐Pt(II) complexes (for example, **44**‐**46**) containing a methionine linker and an Ir‐dppz portion were reported. The complexes can carry an overall 4+ charge and were studied for their DNA binding attributes since the presence of the different Pt(II) moieties could promote additional non‐covalent or covalent interactions with DNA (Figure 27). Analysis of the complexes with the aquated Pt(II) centre (**44**, **45**) suggest both an intercalative interaction from the Ir(III)‐dppz fragment and a covalent (coordinative) interaction between Pt(II) and ct‐DNA. In the case of the Pt(II)‐terpy (**46**) variant the binding constants and supporting data imply almost exclusive mono intercalation via the Ir(III)‐dppz moiety.

**FIGURE 28:**
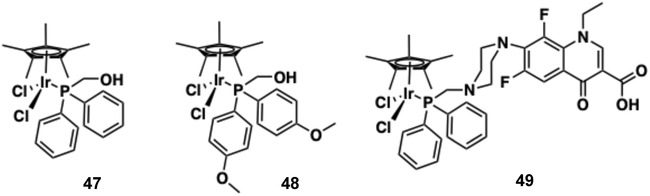
Structures of phosphine coordinated, piano stool Ir(III) complexes **47**–**49**.

Komarnicka and co‐workers investigated the DNA interactions of two half‐sandwich organo‐Ir(III) complexes, **47** and **48** (Figure 28) which incorporate a tertiary phosphine co‐ligand [105]. The biophysical techniques used in the study were competitive fluorescence assays, plasmid DNA gel electrophoresis and computational molecular docking. Fluorescence quenching measurements using EB showed that **47** binds preferentially by intercalation (*K*_*sv*_ = 2.28 × 10^3^ M^−1^), while **48** favoured minor groove binding (*K*_*sv*_ = 2.21 × 10^3^ M^−1^), with methoxy substitution decreasing intercalation and enhancing groove affinity. Electrophoresis revealed that **48** is the more effective cleaving agent, producing single‐strand breaks at 100 μM and double‐strand breaks at higher concentrations, whereas **47** only caused single‐strand nicks above 500 μM. Docking studies confirmed that the minor groove is the most likely binding site, with binding free energies of −1.88 to −2.16 kcal mol^−1^. The same group also explored analogues that integrate fluoroquinolone antibiotic functionalised aminophosphine ligands (e.g. complex **49**) which show DNA interaction mainly through minor groove binding [190].

**FIGURE 29:**
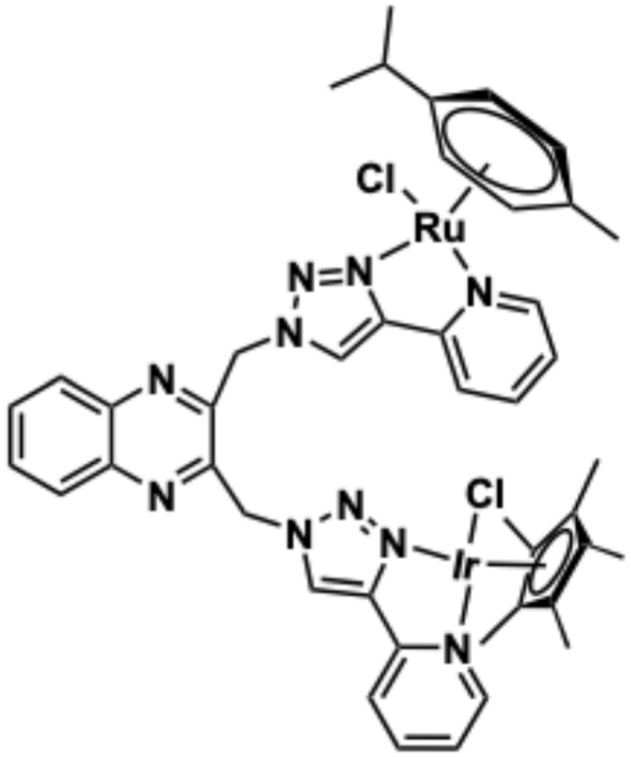
Bimetallic structure of complex **50**.

Heterometallic Ir(III)/Ru(III) species have been developed based upon interlinked piano stool complexes. Paira and co‐workers reported the design of a Y‐shaped mixed metal complex **50** (Figure 29) and compared it to the homometallic Ru(III) analogue [191]. Both complexes are coordinated by a 2‐pyridinyl‐triazolyl chelate linked to a 2,3‐disubstituted quinoxaline bridge. Complex **50** binds to DNA primarily through intercalation, with a binding constant of 0.44 × 10^4^ M^−1^ and an apparent binding constant of 2.8 × 10^6^ M^−1^, demonstrating stronger affinity than the homometallic analogue. DNA binding was confirmed through UV–vis titrations (showing hypochromism), EB displacement assay, viscosity enhancement, and supported by computational molecular docking studies. Interestingly, complex **50** demonstrates cytoselectivity, and mitochondrial localisation, establishing it as a potential theranostic candidate.

## 10 DNA Binding Behaviour of Peptide Functionalised Ir(III) Species

Metal complexes that are functionalised with peptides have attracted significant attention over the years due to the advantages that bioconjugation can impart. Enhanced biocompatibility and improved aqueous solubility are obvious motivations, but rational targeting of metal complexes for therapeutic and diagnostic applications directly benefit from the use of peptide conjugation [192, 193, 194, 195]. Both cyclometalated and half‐sandwich Cp* Ir(III)‐peptide bioconjugates have been reported [ 196] in the context of (targeted) cell imaging using confocal fluorescence microscopy [197] and anticancer agents, respectively [198]. Commonly, binding to biological targets (e.g. receptors, antibodies, proteins) by Ir(III)‐peptide bioconjugates are described, but there are apparently very few reports that explicitly study interactions with DNA.

**FIGURE 30:**
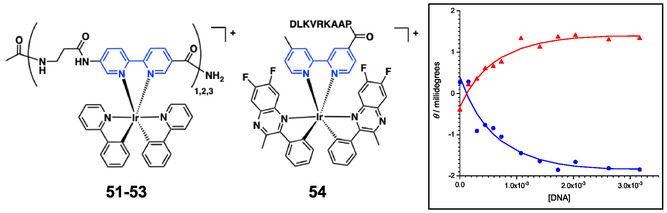
Structure of peptide functionalised Ir(III) complexes **51–54**. Shown inset: CD spectroscopy titration of **54** with fish sperm DNA 373 nm (•), 493 nm (▴).

For example, mono‐, di‐, and trinuclear cyclometalated Ir(III) organometallopeptides (**51–53**) were developed, and their octa‐arginine conjugates were shown to display very high DNA binding affinity and sequence selectivity (Figure 30). Bioconjugation introduced more positive charges and thus enhances DNA affinity, which was demonstrated through different luminescence (*λ*_em_ = 620 nm due to ^3^MLCT/^3^LLCT of the [Ir(ppy)_2_(bipy)]^+^ luminophores) titrations; the trimetallic Ir(III)‐peptides revealed a binding constant of the order 10^8^ M^−1^, which is very high for a non‐intercalating, minor groove binder [199]. In contrast, our own studies have shown that functionalising a photoluminescent Ir(III) complex (**54**) with a nuclear localisation sequence peptide (PAAKRVKLD) leads to low affinities for DNA (ca. 10^3^ M^−1^ shown through UV–vis titrations) despite clear evidence for nuclear localisation in live cell imaging [200].

As noted earlier, the relative lability of chloride in [(*η* ^5^‐Cp*)Ir(N ^ N)Cl] complexes can allow controllable ligand exchange. In 2002, Sheldrick and co‐workers exploited this reactivity to give [(*η* ^5^‐Cp*)Ir(dppz)(L)]^n+^ complexes with sulphur coordinated amino acids and peptides (L) [201]. The series of complexes contained different amino acid constituents (including methionine and cysteine) giving different overall charges (+1 to +3) and charge distributions in phosphate buffer at pH 7.2. Studies with ct‐DNA (UV–vis titrations with data fits using the Bard and Thorp model, and thermal denaturation studies) suggested an intercalative binding mode with an affinity up to ca. 10^6^ M^−1^ for the tricationic variant.

## 11 Conclusions

Iridium(III) coordination complexes, especially those with tunable photophysical attributes, provide a rich molecular framework through which to investigate DNA‐binding phenomena. The physical (including charge, solubility, and sterics), electronic, and redox properties of the complexes enable a suite of biophysical techniques to be utilised for this purpose, combining experimental and computational approaches. The discussion in this review has encompassed three types of Ir(III) complex (half‐sandwich, ‘piano stool’; octahedral polypyridine; octahedral cyclometalated) revealing distinctive approaches to their design as DNA binders. It also reveals remarkable opportunities for targeting dual DNA binding, cytotoxic/phototoxic and/or bioimaging capability, which is critical when considering prospective agents for diagnosis and treatment of disease. The optimisation of complex functionality is achieved through rational ligand design (of which there are myriad and almost limitless variants) and is demonstrably important in DNA binding probes based upon Ir(III) species. Taken together with developments in phototherapeutics and the ability to tune the optical properties of Ir(III) complexes towards the near‐IR window, ligand‐complex Ir(III) architectures should attract the attention of diverse research communities interested in the utility of metal complexes in these disciplines.

## Conflicts of Interest

The authors declare no conflicts of interest.
